# Introducing a New Breed of Wine Yeast: Interspecific Hybridisation between a Commercial *Saccharomyces cerevisiae* Wine Yeast and *Saccharomyces mikatae*

**DOI:** 10.1371/journal.pone.0062053

**Published:** 2013-04-17

**Authors:** Jennifer R. Bellon, Frank Schmid, Dimitra L. Capone, Barbara L. Dunn, Paul J. Chambers

**Affiliations:** 1 The Australian Wine Research Institute, Glen Osmond, Australia; 2 The University of Adelaide, School of Agriculture and Wine, Glen Osmond, Australia; 3 Department of Genetics, Stanford University, Stanford, California, United States of America; Institut de Genetique et Microbiologie, France

## Abstract

Interspecific hybrids are commonplace in agriculture and horticulture; bread wheat and grapefruit are but two examples. The benefits derived from interspecific hybridisation include the potential of generating advantageous transgressive phenotypes. This paper describes the generation of a new breed of wine yeast by interspecific hybridisation between a commercial *Saccharomyces cerevisiae* wine yeast strain and *Saccharomyces mikatae*, a species hitherto not associated with industrial fermentation environs. While commercially available wine yeast strains provide consistent and reliable fermentations, wines produced using single inocula are thought to lack the sensory complexity and rounded palate structure obtained from spontaneous fermentations. In contrast, interspecific yeast hybrids have the potential to deliver increased complexity to wine sensory properties and alternative wine styles through the formation of novel, and wider ranging, yeast volatile fermentation metabolite profiles, whilst maintaining the robustness of the wine yeast parent. Screening of newly generated hybrids from a cross between a *S. cerevisiae* wine yeast and *S. mikatae* (closely-related but ecologically distant members of the *Saccharomyces* sensu stricto clade), has identified progeny with robust fermentation properties and winemaking potential. Chemical analysis showed that, relative to the *S. cerevisiae* wine yeast parent, hybrids produced wines with different concentrations of volatile metabolites that are known to contribute to wine flavour and aroma, including flavour compounds associated with non-*Saccharomyces* species. The new *S. cerevisiae* x *S. mikatae* hybrids have the potential to produce complex wines akin to products of spontaneous fermentation while giving winemakers the safeguard of an inoculated ferment.

## Introduction

The *Saccharomyces* sensu stricto complex is a group of closely related yeast species that can mate to form interspecific hybrids. Natural *Saccharomyces* interspecific hybrids have been isolated from various fermentation environs. The lager yeast *Saccharomyces pastorianus,* ( syn *Saccharomyces calsbergensis*), first described in 1883 by Emil Christian Hansen, is a stable, natural hybrid between *S. cerevisiae* and *Saccharomyces eubayanus* [1], [2], [3]. A small number of wine yeast and cider yeast strains have also been identified as natural interspecific hybrids between the *Saccharomyces* species, *S. cerevisiae*, *S. bayanus* and *S. kudriavzevii* [1], [4], [5], [6]. Although no natural hybrids between *S. cerevisiae* and *S. mikatae* have been reported to-date, two ale strains have been shown to contain a small (4.5 kb) *S. mikatae* introgressed non-coding region corresponding to the right end of chromosome VI [7].

Here we describe for the first time, the generation of an interspecific hybrid between a commercial *S. cerevisiae* wine yeast strain and *S. mikatae*, a species not previously associated with alcoholic fermentation and isolated only from soil and decaying leaf litter [8], [9]. Although members of the *Saccharomyces* sensu stricto group are considered to be closely related yeast, DNA sequence variation between the most distantly related species within this group corresponds roughly to that between man and mouse [10].

The driver for this work comes from a desire in the wine industry to develop novel yeast strains that bring greater complexity to wine than strains currently available to the industry. Winemakers grapple with many issues when deciding their winemaking practices including consistency in wine style and quality across vintages, and dealing with the risk of spoilage by indigenous microorganisms. With these concerns in mind, the process of inoculating grape must with a single, proven commercial strain, (typically *S. cerevisiae*), has become the backbone of modern winemaking. Commercial yeast strains have robust growth properties in demanding conditions (low pH, osmotic stress due to the initial high sugar concentration of grape must and accumulation of alcohol in the later stage of fermentation), and out-compete indigenous microorganisms to carry out fermentation in a timely manner while producing reliable, quality wines.

Whilst there are indications that contributions from the many different indigenous microorganisms in uninoculated spontaneous fermentations build a more complex palate structure and greater diversity of flavour profiles [11], the unpredictable nature of spontaneous fermentations leads many winemakers to prefer an inoculation regime where the microorganism population is controlled. One approach to reaping the benefits of spontaneous fermentations while minimising risk of spoilage is to use inoculations with multiple *S. cerevisiae* wine yeast strains or *S. cerevisiae* and non-*Saccharomyces* strains. However, studies show that growth variability can occur between strains with unpredictable results [12], [13], [14], presumably due to the differential fitness of strains in highly variable grape juice compositions. A strategy that avoids the problem of competition between strains is to hybridise the genomes of two different species, generating an interspecific hybrid yeast strain capable of producing a wide range of flavour-active metabolites.

Mating in *Saccharomyces* spp. is typically between haploid cells of the opposite mating type (**a** and **α**). For the purpose of generating novel interspecific wine yeast however, it was decided to retain the full complement of the wine yeast parent diploid genome in the new hybrids; diploid wine yeast were therefore mated with haploid *S. mikatae*. This can be achieved because diploid *S. cerevisiae* cells can undergo a low frequency (1×10^−6^) mating type switch that results in a diploid cell homozygous at the mating type locus, **a**/**a** or **α**/**α** [15]. These homozygotes can enter the mating pathway and conjugate with a cell of the opposite mating type, leading to the generation of polyploid interspecific hybrids.

Hybrid progeny from rare matings between *S. cerevisiae* and *S. mikatae* were screened for fermentation traits and their wines analysed for basic fermentation chemistry. Subsequently, two hybrid strains were selected for further study and the wines produced by these hybrids and the parent wine yeast were analysed for volatile and solvent extractable fermentation products as well as phenolic content. The genetic stability of these two hybrid strains was also assessed.

## Materials and Methods

### Yeast strains and media

Parental strains: *S. cerevisiae* AWRI838 (an isolate of the commercial wine yeast strain EC1118), *S. mikatae* type strain NCYC2888 (designated AWRI1529); a diploid, prototrophic, heterozygous and homothallic wild yeast strain [16]; and hybrid strains generated from this study, CxM1 – CxM5 (CxM1 designated AWRI2526), were grown in YEPD medium (1% w/v yeast extract, 2% w/v peptone, 2% w/v glucose) with shaking (100 rpm) at 25°C for one day. Mitochondrial mutants of AWRI838 were generated by ethidium bromide mutagenesis [17]. Ploidy control strains for fluorescence flow cytometry analysis were: BY4742 mat alpha, haploid and BY4743, diploid, (Euoroscarf®) and 53–7 tetraploid [18].

### Generation of interspecific hybrid yeast

Rare-mating was used for interspecific hybridisations as described previously by [17]. Cells from the cross were washed in sterile water and plated onto YEP-glycerol-ethanol selection medium (1% w/v yeast extract, 2% w/v peptone, 3% w/v glycerol, 14% v/v ethanol, 2% w/v agar) and incubated at 22°C.

### PCR confirmation of hybrids

DNA was isolated from yeast using mechanical breakage with glass beads [19]. Genomic DNA was used as template for PCR analysis, with amplification using the rDNA Internal Transcribed Spacer primer pair ITS1/ ITS4 (**Table 1**) followed by digestion with Restriction Enzyme *Hae*III ; fragments were resolved on a 3% w/v agarose gel [20].

**Table 1:** Primer sets and restriction endonucleases used to generate species-specific chromosomal markers.

Primer	R/E	Sequence
ITS1	*Hae*III	TCCGTAGGTGAACCTGCGG
ITS4		TCCTCCGCTTATTGATATGC
ScSm IL	*Alu*I	ATTTCTGAATCGTACTGTGCG
		ACCTCGATGACATTGTCGGAT
ScSm IIR	*Taq*I	CGCATTGGGAAGAATTAGTGG
		TCGTCAACCTGTAAGGAATCG
ScSm IIIR	*Taq*I	TGGCTTTGGAACCTATTGATT
		ATGAAGATTCCGTCATGGAGG
ScSm IVR	*Mse*I	TTTTTGTTCCTGCAGATTTTG
		ACCTGGTAGGGCCCATGAT
ScSm VL	*Taq*I	TTTCAAGTCACTGACGTGGCA
		CATCTGCGATTTCTTGGCAA
ScSm VR	*Taq*I	TTCCGCACTATTATCGCAGA
		TTTGTGCAATAGTGGGTGAGG
ScSm VIL	*Hae*III	GGTGCTGCATTCTGGGAAA
		GGCATCAAACATTTGCTGTG
ScSm VIIL	*Taq*I	TCCATTGGGTTTCACCTTTTC
		AGCAGCAATACCACAAACGGA
ScSm VIIIR	*Taq*I	TCGTTTTGGACACAGGAAAG
		GGAAACCTTTTCGTAGCGTGA
ScSm IXL	*Rsa*I	AACAAGGGGAACAGTCTGTCA
		AGAACACAGCAATGTTCCCA
ScSm XL	*Hae*III	CACTCCAATCAACGCTGAAAA
		TAAATGACCTGGGACATCCA
ScSm XR	*Taq*I	CGTTTATTGTGCCGAGCTTA
		TTGGATATGTCAAAGCCAGG
ScSm XIL	*Taq*I	AAATGCAGTGAACGATCCACG
		AGATGATGGCCAGTATGCAA
ScSm XIIL	*Hae*III	CGGTGAAGGTGCCAAATAC
		AGCAGCATGAATACCCCAGTT
ScSm XIIR	*Mse*I	ATTGGCTCGGTACCCCTTT
		TGCCCACATCTGAGACAAAA
ScSm XIIIR	*Hae*III	TGGACTCCAATGTATTGGACG
		ATGTGGAAATCTTGGCCCTT
ScSm XIVL	*Hae*III	TTTAGCGTGGACGATGATCC
		CCCAATTGTAGAATTGCTGC
ScSm XIVR	*Hae*III	AATGGATTTACGCGGCAATAG
		GGCAGTTTGATTTCTAGCGGT
ScSm XVR	*Taq*I	CAAGGCCAAGATGATGAAGA
		TTCTTTCCCACGTTTGGAAG
ScSm XVIL	*Hae*III	TTCTCCAATCATTGCCACCT
		TTGGCGTTGAAAGATCTCCA
ScSm XVIR	*Hae*III	AAATTCTGGTAATCCATGGGA
		TTCAACCATCTCCTTGGTGTG

### Genomic stability of hybrid isolates

To verify that hybrid strains retained the genomes of both parents following grape juice fermentation, end-of –fermentation isolates (50 colonies) from each of the triplicate hybrid ferments were analysed with the ITS1/4 primer set and *Hae*III Restriction Enzyme. Subsequently, 50 isolates of each hybrid from one of the triplicate end-of–ferment samples were investigated for genome stability using PCR/RFLP targeting at least one arm of each chromosome; 21 primer sets in total (**Table 1**). Primers were designed with homology to both the sequenced *S. cerevisiae* laboratory strain S288c and *S. mikatae* strain IFO 1815 using the primer design tool accessed from the *Saccharomyces* Genome Database website (http://www.yeastgenome.org/cgi-bin/seqTools). The above ITS PCR program was used except for the annealing temperature that was lowered to 50°C to accommodate a maximum of one missmatched base to either species’ DNA sequence in the mid region of a primer. Amplified fragments were then digested with restriction endonucleases to generate species-specific banding patterns. Resultant fragments were resolved on a 3% w/v agarose gel.

### Array-Comparative Genome Hybridisation (a-CGH) of *S. cerevisiae* x *S. mikatae* hybrid AWRI 2526 and parent strains

A**-**CGH hybridization and data analysis was performed as described in [7] using custom microarrays manufactured by Agilent Technologies containing 60-mer oligonucleotides designed to the *S. cerevisiae* S288c and *S. mikatae* IFO 1815 genomes. After quality filtering, data representing 24,000 probes evenly spaced across the *S. cerevisiae* genome and 1,600 probes evenly spaced across the *S. mikatae* genome were used for further examination and analysis.

### Fluorescence flow cytometry analysis to determine ploidy of putative interspecific hybrids

Strains were grown in YEPD for five days to late stationary phase and fixed in 70% ethanol. A sample of 1×10^6^ cells was processed by washing with sodium citrate (50 mM), RNA was removed with RNAse A and the sample was stained with propidium iodide (2 µg/ml). Cells were analysed using a FACSCalibur (Becton Dickinson, Australia) instrument equipped with a 15 milliwatt air-cooled argon-ion laser emitting at 488 nm. Cells were detected at 585/42 nm (FL2) using BD FACSFlow™ sheath fluid and fluorescence plotted to a linear scale.

### Phenotypic assessment of interspecific hybrids

Ethanol and glucose tolerances were determined as described by [17]. To determine sensitivity to different growth temperatures, standard YEPD plates were incubated at 37°C (high temperature stress), 4°C (low temperature stress) or 22°C (non-stress control). Strains were grown to stationary phase in liquid YEPD (2 days) and 5 µl of 10 fold serial dilutions were spotted to plates.

### Grape juice fermentation

Hybrid strains were screened for robust fermentation properties in filter sterilised Chardonnay juice (total sugar, (glucose and fructose) 250 g/L, yeast assimilable nitrogen 227 mg/L, pH 3.01) sourced from The Yalumba Wine Company (South Australia). All strains were initially grown in YEPD for 2 days and then acclimatised by 2 days growth in ½ X Chardonnay grape juice medium (diluted with sterile water), shaking, for 2 days. Triplicate 100 ml fermentations were carried out in Chardonnay juice at 22°C. Juice was inoculated at 2×10^−6^ cells per ml and fermentations carried out in conical flasks fitted with water traps, shaken at 150 rpm. Cell growth was measured using Optical Density (absorption at 600 nm) while utilisation of sugar was measured by Refractive Index using an Atago® Palette Digital Refractometer. Triplicate fermentations were sampled in duplicate for chemical analyses.

### Wine chemical analysis

Concentrations of residual sugars (glucose and fructose), ethanol, glycerol, and acetic, succinic, malic, lactic, citric and tartaric acids, were determined by HPLC using a Bio-Rad HPX-87 column [21].

### Targeted volatile fermentation products analysis

Samples were analysed using stable isotope dilution combined with gas chromatography/mass spectroscopy (GC/MS) [22]. Wine samples were prepared in 2 dilutions, 1/20 and 3/10, with Model Wine (11% ethanol, 10% potassium hydrogen tartrate, pH adjusted with tartaric acid to 3.1). Analysis was performed on an Agilent 7890A gas chromatograph equipped with Gerstel MPS2 multi-purpose sampler and coupled to an Agilent 5975C VL mass selective detector. Instrument control and data analysis were performed with Agilent ChemStation software.

### Solvent-extractable volatile chemical analysis

**A** 10 mL wine sample was extracted with 3 mL of Pentane:ethyl acetate (2∶1) and the organic layer was then transferred to a (2 mL) vial for GC/MS analysis. Samples were analyzed with an Agilent 6890A gas chromatograph fitted with a Gerstel MPS2 autosampler and coupled to an Agilent 5973N mass spectrometer. The gas chromatograph was fitted with a 60 m J & W DB-Wax fused silica capillary column (0.25 mm i.d., 0.25 µm film thickness). The auto sampler was fitted to a liquid injector operated in fast liquid injection mode with a 10 µL syringe fitted. The carrier gas was helium and the flow rate was 1.7 ml/min. The oven temperature started at 50°C, was held at this temperature for 1 min., then increased to 240°C at 4°C/min. and held at this temperature for 10 min. The injector was held at 200°C and the transfer line at 240°C. The sample volume injected was 2 µL and the splitter, at 33∶1, was opened after 36 sec. Fast injection was performed in pulse splitless mode with an inlet pressure of 45.0 psi maintained until splitting. The liner was borosilicate glass with a plug of resilanised glass wool (2–4 mm) at the tapered end to the column. Positive ion electron impact spectra at 70 eV were recorded in the range *m/z* 35–350 for scan runs. The identification of compounds was performed by comparison of their retention time and of mass spectra with that of the mass spectral data stored in database libraries; Australian Wine Research Institute, Wiley 275 and NB 275K.

### Analyses of wine polyphenolics

Wine samples were scanned in the range 600 nm to 240 nm using a Varian CARY 300 UV-Visible Spectrophotomer. Total Phenolics and Total Hydroxycinnamic Acids were determined spectrophotometrically using the absorbance at 280 nm and 320 nm respectively (10 mm pathlength). Total hydroxycinnamates were quantified as ‘caffeic acid equivalents’, CAE (mg/L) from the spectral reading at 320 nm:
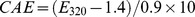



Total Flavonoid Extracts were determined spectrophotometrically as absorption units (a.u.) at 280 nm (10 mm path), taking into account the contribution of non-phenolics and total hydroxycinnamates by use of the formula:



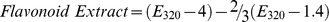

[23]

The values 4 and 1.4 are statistically based correction factors for non-phenolics at 280 and 320 nm respectively; and the fraction 2/3 refers to the ratio of hydroxycinnamate absorbance at 280 to that at 320 nm.

Total flavonoids were quantified as ‘catechin equivalents’, CE (mg/L), from Flavonoid Extract (FE) a.u.:



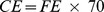

[24]

### Statistical Analyses

A one-way analysis of variance (ANOVA) and Student’s t-test (p<0.05), were used to determine significant differences between wines.

## Results

### Generation and phenotypic characterisation of novel *S. cerevisiae* x *S. mikatae* hybrids

**Figure 1:**
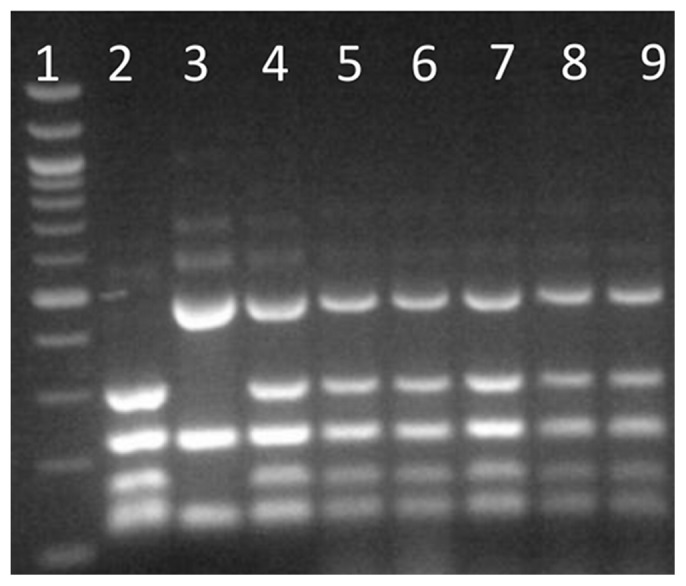
Genetic confirmation of cell hybridization by rDNA ITS PCR-RFLP. Lane 1 100 bp ladder, lane 2 AWRI838, lane 3 NCYC2888, lane 4 DNA from both parents, lanes 5–9 Hybrids CxM1-5.

**Figure 2:**
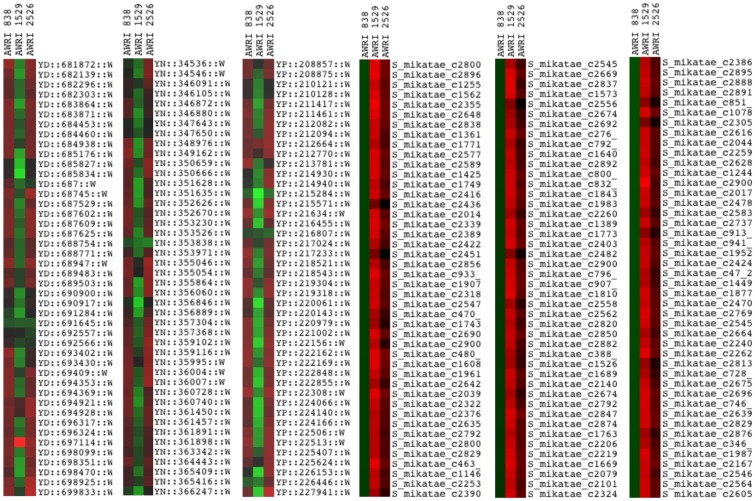
Sample sets of array-CGH data for parents and hybrid strain CxM1. Within each panel of microarray data, each column contains the a-CGH data for a given strain while each row corresponds to a probe for a chromosomal location. The leftmost three panels show the data for probes to the S. cerevisiae genome, located on chromosome V (“YD’ followed by chromosome coordinate), XIV (‘YN”), and XVI (”YP”); the rightmost three panels show data for probes to various regions (contig “c” followed by contig number) of the *S. mikatae* genome. 838 is the *S. cerevisiae* parent strain, AWRI 1529 is the *S. mikatae* parent strain NCYC2888, and AWRI2526 is the hybrid strain CxM1. Red hybridisation intensities for a probe indicate the presence of that species’ genome region, while green hybridisation intensities indicate the absence of that species’ genome region. The reduced intensity of *S. mikatae* probes in the hybrid dataset indicates a reduced *S. mikatae* ploidy level relative to *S. cerevisiae*, within the hybrid genome.

Rare mating of the diploid *S. cerevisiae* wine yeast strain, AWRI838, with spores of *S. mikatae* strain NCYC2888, produced five interspecific hybrid colonies (CxM1 - CxM5). Species specific PCR–RFLP of target rDNA confirmed that both parental genomes were present in these hybrids (**Figure 1**). Array-Comparative Genome Hybridisation (a-CGH) was performed on one hybrid strain (CxM1 designated AWRI2526) and the two parental strains. The microarray generated from 1600 *S. mikatae* specific probes and 24,000 *S. cerevisiae* specific probes further confirmed that this hybrid strain’s genome contained an entire chromosome set from each parent, and appeared to confirm the expected 2∶1 *S. cerevisiae*:*S. mikatae* ploidy ratio (**Figure 2**). (Average *S. mikatae* probe intensity was 2.244 for *S. mikatae* parent NCYC2888 and 0.935 for hybrid strain CxM1, indicating a reduction of *S. mikatae* genome in the hybrid strain from diploid to haploid.)

**Figure 3:**
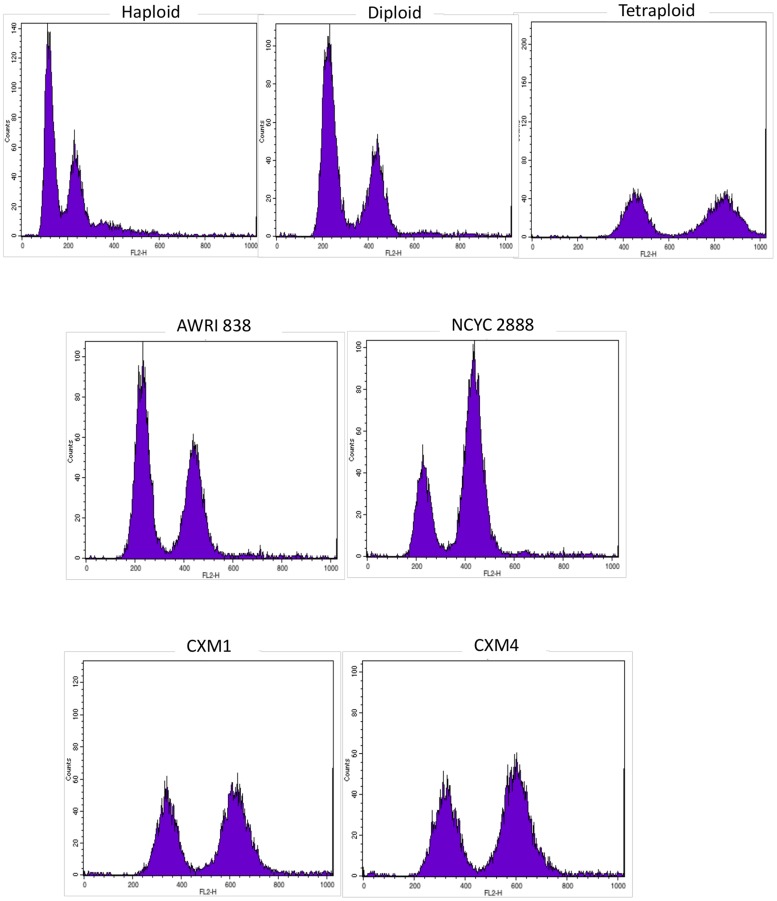
Fluorescence flow cytometry analysis. Top row left to right; Control ploidy strains BY4742 (haploid), BY4743 (diploid) and 53–7 (tetraploid). Middle row left to right; Parent strains AWRI838 and NCYC2888. Bottom row left to right; Hybrid strains CxM1 and CxM4.

To determine ploidy levels of hybrids, relative genomic DNA content was assessed by fluorescence flow cytometry analysis using linear plots of cell fluorescence. All cultures generated dual peaks of fluorescence, with the second peak attributed to cells undergoing DNA synthesis. Diploid and tetraploid control strains were easily distinguishable with non-dividing cells giving peaks respectively of approximately double and quadruple fluorescent levels of the haploid strain. Parental yeast strains, AWRI838 and NCYC2888, were confirmed as diploids while all hybrid strains gave fluorescent peaks equivalent to a triploid genome complement (**Figure 3**).

**Figure 4:**
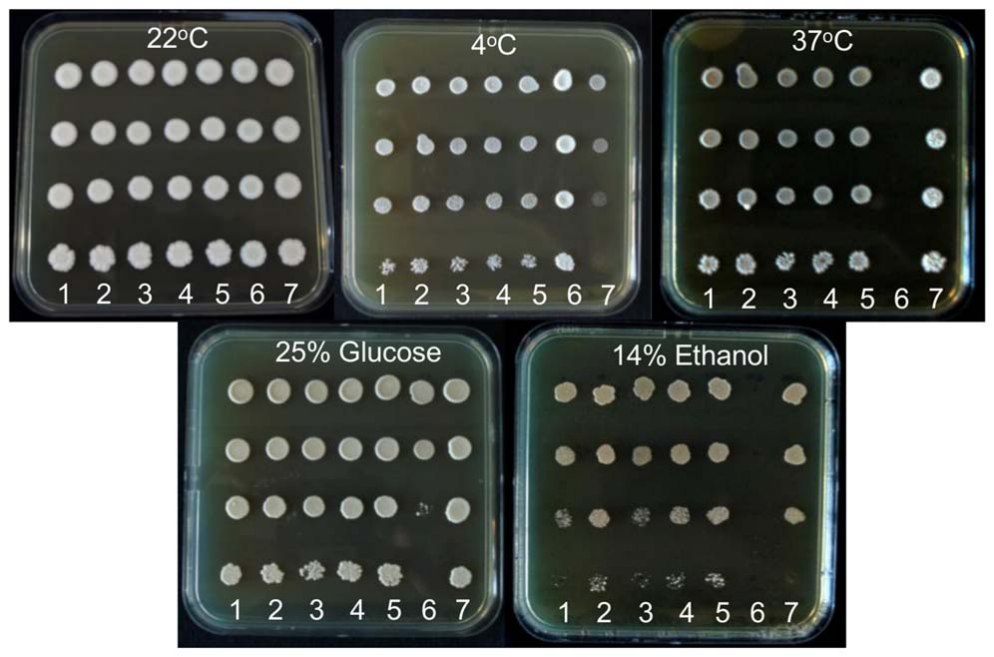
Phenotypic assessment assay plates. Top row plates left to right; YEPD at temperatures 22°C, 4°C and 37°C. Bottom row plates left to right; YEP 25% glucose, YEPD 14% ethanol. Strains are plated in columns at 10 fold serial dilutions from top to bottom; columns 1–5 CxM5-CxM1 in descending order, column 6 NCYC2888, column 7 AWRI838.

Both parental and all hybrid strains were able to grow well on YEPD plates at the non-selective temperature of 22°C. The *S. cerevisiae* parent strain showed strong growth in all conditions except low temperature (4°C). On the other hand, the *S. mikatae* parent grew well at 4°C, poorly on high glucose and was non-viable at both 37°C and high ethanol (14%) concentration. All five hybrid strains were able to grow well in all conditions; high and low temperatures, high glucose and high ethanol concentrations. In fact, a small amount of hybrid vigour is evident at high ethanol concentrations, with three of the hybrid strains showing greater ethanol-tolerance than their *S. cerevisiae* parent (**Figure 4**).

### Grape juice fermentation and basic chemical analyses of wines

**Figure 5:**
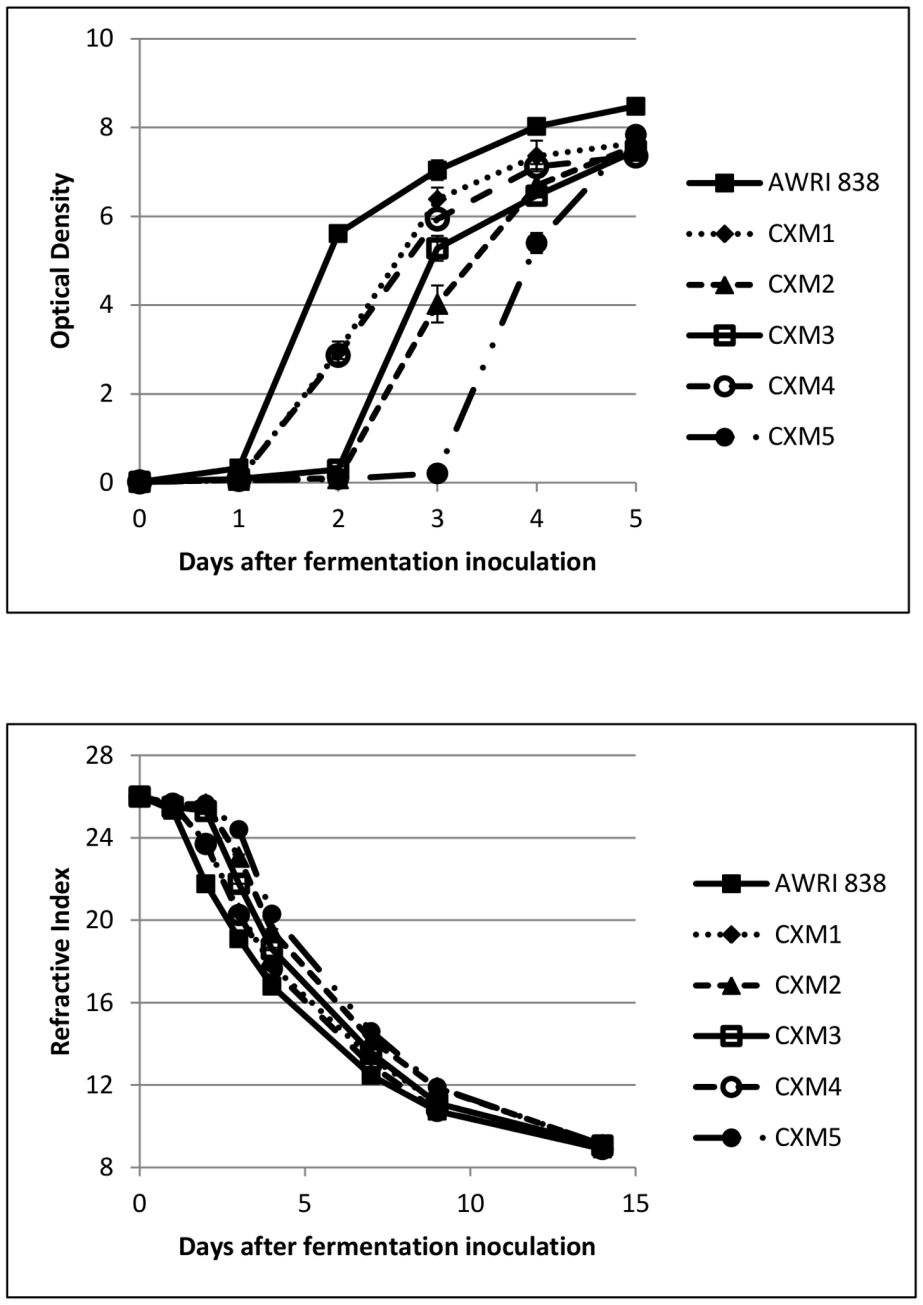
Grape juice fermentation profile of AWRI 838 and hybrid strains CxM1-CxM5. Figure 5a. (top) Cell growth during fermentation as determined by Optical Density. Data points are presented with error bars. Figure 5b. (bottom) Sugar utilisation during fermentation as determined by Refractive Index. Data points are presented with error bars.

All five hybrid strains completed fermentation in reasonable time. However, several of them found this medium challenging, with no discernible increase in cell number until the third (CxM2 and CxM3) or fourth (CxM5) day, whereas the wine yeast parent strain and two hybrid strains (CxM1 and CxM4) showed strong growth after the first day following inoculation (**Figure 5a**). No fermentation profile is shown for the *S. mikatae* parent strain as it was unable to grow in Chardonnay juice. Refractive index measurements (an indication of sugar utilisation) showed that the wine yeast parent and the faster-growing of the hybrid strains (CxM1 and CxM4) consumed sugars at a higher rate than other hybrids, and with a shorter growth lag-time (**Figure 5b**). Although final R.I. measurements were similar for all ferments, wines produced by hybrid strains CxM2 and CxM3 had detectable residual fructose (**Table 2**). Wine produced by hybrid strain CxM2 contained 4.5 g/L of fructose, a level considered by winemakers to be too high for the wine to be classed as ‘Dry’, the maximum for this is less than of 4.0 g/L residual sugar (European Union Commission Regulation EC 753/2002). CxM3 produced wines with the lowest concentration of ethanol (15.8%) while this hybrid strain was also one of the higher glycerol producers, 12.1 g/L compared to the wine yeast parent (16.3% ethanol and 9.6 g/L glycerol). Four of the five hybrids produced wines with no detectable acetic acid; CxM4 produced 0.22 g/L acetic acid, approximately 50% of the parent level (0.41 g/L). In general, the hybrid strains produced wines with equivalent, or slightly higher, levels of citric, malic and succinic acid (97–120%), much higher levels of lactic acid (125–185%) and lower levels of tartaric acid (85–95%).

**Table 2:** Fermentation chemistry analysis of wines using HPLC.

	AWRI838	CxM1	CxM2	CxM3	CxM4	CxM5
Glucose *	<0.1	<0.1	<0.1	<0.1	<0.1	<0.1
Fructose *	<0.1 c	<0.1 c	4.51±0.07 a	2.23±0.108 b	<0.1 c	<0.1 c
Ethanol ≠	16.3±0.06 a,b	16.4±0.02 a	16.1±0.09 b,c	15.8±0.06 c	16.1±0.02 a,b,c	16.4±0.01 a
Glycerol *	9.6±0.03 d	11.1±0.01 c	11.6±0.03 b	12.1±0.07 a	11.4±0.06 b,c	12.1±0.10 a
Acetic acid *	0.41±0.01 a	<0.1 c	<0.1 c	<0.1 c	0.22±0.04 b	<0.1 c
Succinic acid *	4.14±0.01d	4.59±0.01 c	4.53±0.01 c	4.75±0.04 b	4.59±0.03 c	4.85±0.01 a
Malic acid *	2.83±0.02 c	2.98±0.01 b	3.09±0.01 a	2.88±0.01 c	2.81±0.02 c,d	2.75±0.02 d
Lactic acid *	0.32±0.01 c	0.60±0.00 a	0.42±0.01 b	0.59±0.00 a	0.40±0.01 b	0.44±0.02 b
Tartaric acid *	3.12±0.01 a	2.61±0.01 b	2.62±0.01 b	2.71±0.01 b	2.68±0.01 b	2.94±0.08 a,b
Citric acid *	0.12±0.00 d	0.12±0.00 d	0.14±0.00 b	0.15±0.00 a	0.13±0.00 c	0.14±0.00 b

Detection Limit 0.1g/L * g/L, ≠ % v/v Levels not connected by same letter are significantly different (p<0.05).

### Genetic stability of novel *S. cerevisiae* x *S. mikatae* hybrids

**Figure 6:**
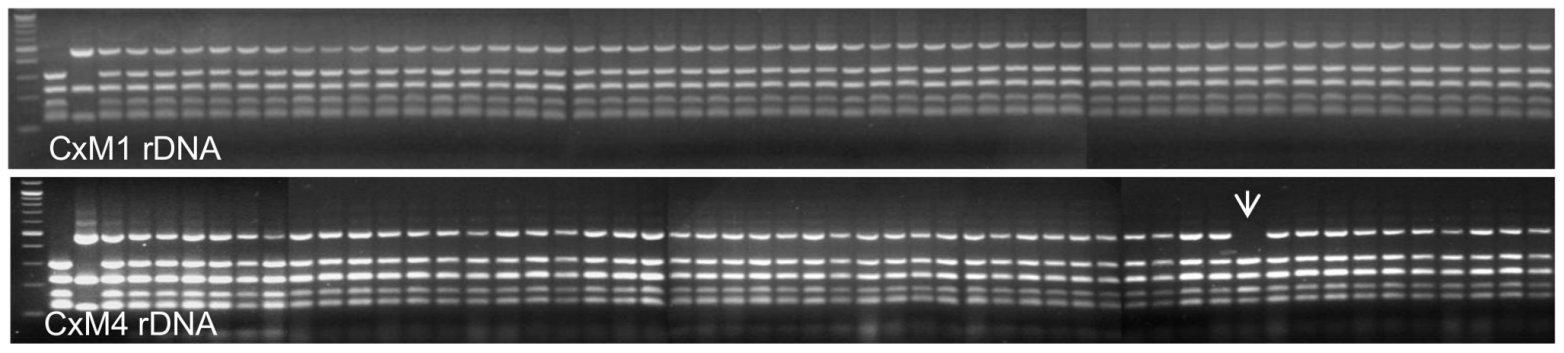
Genetic stability of *S. cerevisiae* x *S. mikatae* hybrids using rDNA ITS PCR-RFLP. Top gel, CxM1 fermentation isolates and bottom gel, CxM4 fermentation isolates. Lane 1 100 bp ladder, lane 2 AWRI838, lane 3 NCYC2888, lane 4 DNA from both parents, lane 5, Hybrid, lanes 6–55 isolates 1–50. Arrow points to isolate with loss of *S. mikatae* rDNA.

**Figure 7:**
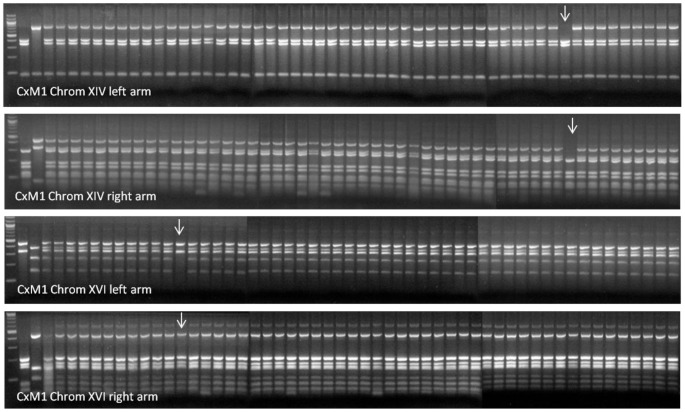
Genetic stability of CxM1 fermentation isolates using chromosomal targeted PCR-RFLP. First gel Chromosome XIV left arm, second gel Chromosome XIV right arm, third gel Chromosome XVI left arm and fourth gel Chromosome XVI right arm. Lane 1 100 bp ladder, lane 2 AWRI838, Lane 3 NCYC2888, lane 4 DNA from both parents, lane 5 Hybrid CxM1, lanes 6 to 55 isolates 1 to 50. Arrows point to isolates with altered chromosomal content.

**Figure 8:**
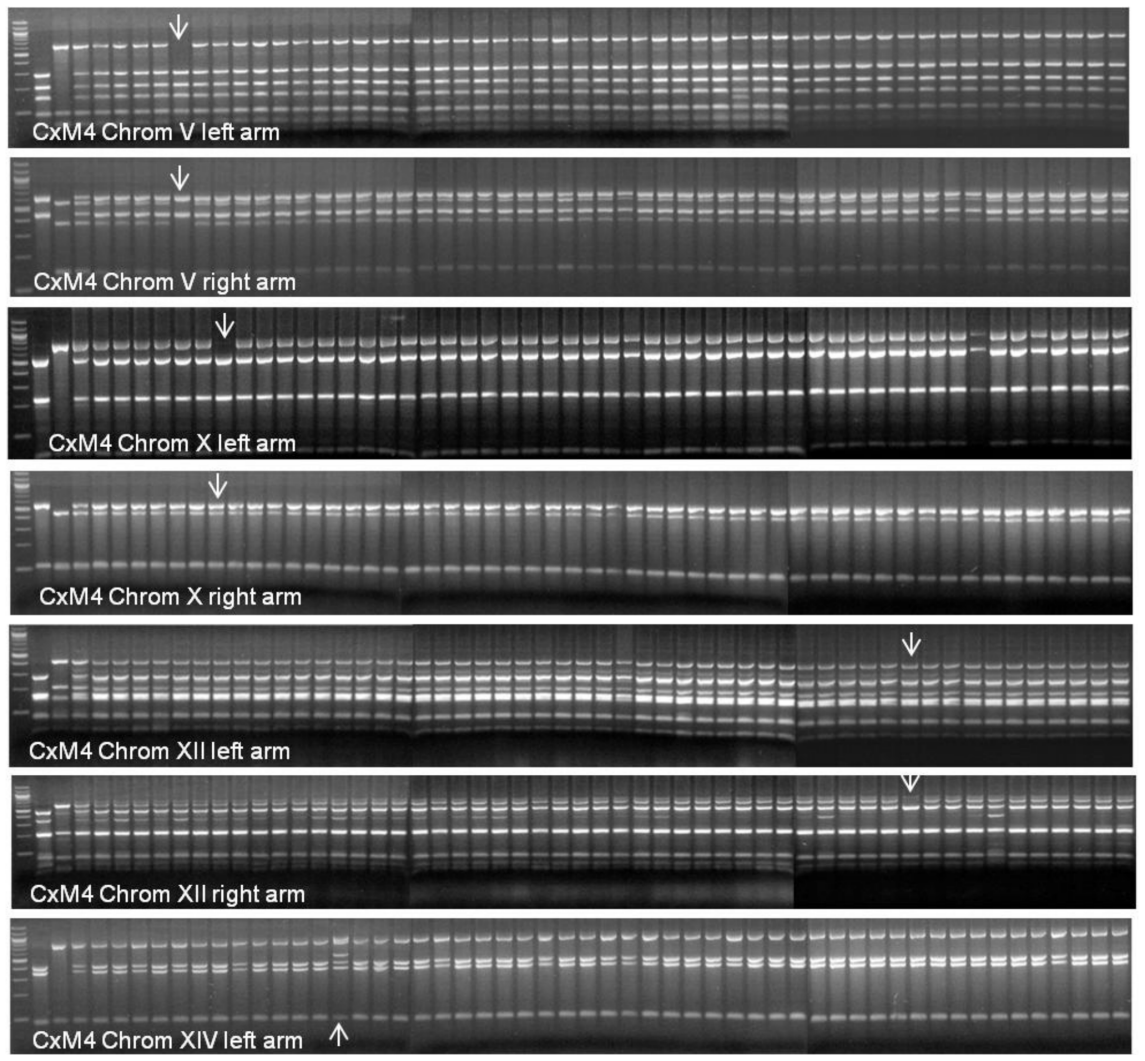
Genetic stability of CxM4 fermentation isolates using chromosomal targeted PCR-RFLP. First gel Chromosome XIV left arm, second gel Chromosome XIV right arm, third gel Chromosome XVI left arm and fourth gel Chromosome XVI right arm. Fifth gel Chromosome XII left arm, sixth gel Chromosome XII right arm, seventh gel Chromosome XIV left arm. Lane 1 100 bp ladder, lane 2 AWRI838, Lane 3 NCYC2888, lane 4 DNA from both parents, lane 5 Hybrid CxM4, lanes 6 to 55 isolates 1 to 50. Arrows point to isolates with altered chromosomal content.

The genetic stability of two hybrid strains considered to have the best fermentation capability, (CxM1 and CxM4), was tested using the same rDNA PCR-RFLP approach as for the confirmation of hybridisation. Fifty end-of-fermentation isolates from each triplicate Chardonnay wine (150 isolates in total for each of the two hybrid yeast strains) were analysed to confirm the retention of rDNA from each species within the hybrid genome. There was no loss of either parental rDNA in isolates of hybrid CxM1 while only one of the 150 CxM4 isolates showed a loss of parental rDNA, with the species specific band of *S. mikatae* missing from the PCR/RFLP pattern (**Figure 6**). Isolates from one of the replicate fermentations of each hybrid strain were further analysed using 21 PCR primer sets targeting at least one arm of all 16 chromosomes. For hybrid strain CxM1, one isolate, (#41), had lost both left and right arms of *S. mikatae* Chromosome XIV and another isolate, (#10) lost only the right arm of *S. mikatae* chromosome XVI (**Figure 7**). There was no sign of loss in the other isolates of this cross. For strain CxM4, four of the 50 isolates showed chromosomal evolution: one isolate (#4) lost of both arms of *S. mikatae* Chromosome V, another isolate (#6) lost only the left arm of *S. mikatae* Chromosome X. A third isolate (#40) lost the right arm of *S. mikatae* Chromosome XII, (which corresponds to this isolate’s loss of rDNA on Chromosome XII observed in the ITS PCR/RFLP), while the fourth isolate (#12) showed a polymorphism at the left arm Chromosome XIV target site (**Figure 8**). No isolate showed loss of DNA on more than one chromosome (**Figure S1**).

Fluorescence flow cytometry analysis on the 50 CxM1 end-of–fermentation isolates showed no discernible loss of ploidy (**Figure S2**).

CxM1 hybrid isolates from the single replicate end-of–fermentation genomic analyses were also screened for the two important fermentation traits of high sugar and high ethanol tolerance. All isolates were able to grow well, however a small reduction of robustness was observed in two of the 50 isolates (**Figure S3**).

### Chemical analysis of fermentation products

Additional triplicate laboratory scale fermentations were carried out in Chardonnay juice using the wine yeast parent, AWRI838, and the two hybrid strains that utilised all sugars during the preliminary fermentation trial, CxM1 and CxM4, without the inclusion of the *S. mikatae* parent strain due to its inability to grow in Chardonnay juice. The resultant wines (all having completed fermentation with < 0.25% residual sugar) were analysed using GC/MS for seventeen volatile fermentation-derived compounds previously determined to be important contributors to the aroma and flavour profile of wines [22]. Additional flavour and aroma compounds were identified by GC/MS scan runs and comparing their mass spectra to libraries of known flavour and aroma compounds.

### Targeted volatile fermentation products analysis

Both hybrid strains showed differences in the concentration of a number of the compounds analysed relative to the wine yeast parent (**Table 3**). Hybrid strain CxM4 displayed the most differences, producing lower concentrations for 13 compounds and a higher concentration for two compounds; 2-phenylethyl acetate and butanol. Three compounds with the undesirable aroma of ‘nail polish’, (ethyl acetate, 2-methylbutanol and 3-methylbutanol), were produced at much lower concentrations by this hybrid, ranging from 40% to 65% relative to the wine yeast parent. However, hybrid CxM4 also produced lower concentrations of nine compounds analysed that comprise ‘fruity’ flavours. In contrast, 2-phenylethyl acetate which elicits a floral aroma, was present at double the concentration compared to the parent. Hybrid CxM1, on the other hand, produced wines with higher concentrations in six compounds, four of which contribute to ‘fruit’, ‘floral’ or ‘perfume’ flavours, while the others contribute ‘fusel’ and ‘nail polish’ aromas. Conversely, another compound having a ‘nail polish’ aroma, (ethyl acetate), was produced at a much reduced level compared to the wine yeast parent (53%).

**Table 3:** Target volatile fermentation products of AWRI 838, CXM1 and CXM4 in Chardonnay wines.

Ethyl esters (µg/L)	Aroma descriptor	AWRI838	CxM1	CxM4
Ethyl acetate	Nail Polish	25036±645 a	13181±4 b	10145±401c
Ethyl propanoate	Fruity	273±27 b	354±22 a	200±5 c
Ethyl 2-methyl propanoate	Fruity	42±6 a	47±4 a	28±1 b
Ethyl butanoate	Fruity	134±12 a	164±12 a	103±3 b
Ethyl 2-methyl butanoate	Sweet fruit	5.03±0.6 b	6.77 0±0.3 a	3.43±0.1 c
Ethyl 3-methyl butanoate	Berry	6.5±0.9 a	7.2±0.3 a	4.4±0.1 b
Ethyl hexanoate	Green apple	230±18 a	235±17 a	140±2 b
Acetates (µg/L)				
2-Methyl propyl acetate	Banana, fruity	16.8±2.1 a	17.6±1.8 a	11.7±0.3 b
2-Methyl butyl acetate	Banana, fruity	31.4±1.2 a	42.6±4.7 a	22.4±0.7 b
3-Methyl butyl acetate	Banana	577±56 a	657±62 a	450±16 b
2-Phenyl ethyl acetate	Floral	196±56 b	389±50 a	394±18 a
Hexyl acetate	Sweet perfume	10.1±0.4 b	13.8±1.4 a	8.0±0.4 c
Alcohols (µg/L)				
2-Methyl propanol	Fusel, spirituous	40871±556 a	41559±2927 a	42856±718 a
Butanol	Fusel, spirituous	1107±6 b	1321±52 a	1283±8 a
2-Methyl butanol	Nail polish	6525±153 b	8892±307 a	4344±340 c
3-Methyl butanol	Harsh, nail polish	8249±1126 a	7882±475 a	3587±98 b
Hexanol	Green, grass	3059±410 a	2594±84 a	2482±36 a

Levels not connected by same letter are significantly different (p<0.05).

### Solvent-extractable volatile chemical analysis

A total of 27 compounds were identified in the solvent-extractable portion of the Chardonnay wines; compounds such as ethyl esters, acids, phenols and alcohols, while three compounds remain un-identified (**Table 4**). Peak area was used as an indication of relative compound concentration between wine samples. Of the 30 compounds, 18 showed different concentrations in the hybrid yeast-made wines relative to the parent yeast-made wines, with thirteen compounds increasing in level and five compounds decreasing. Nine compounds displayed a two-fold (or more) increase; compounds contributing sweet attributes such as β-phenyl ethanol (‘rose’), 9-decenoic acid (‘fruity’) and 3-hydroxy-4-phenyl-2-butanone (‘caramel’), along with compounds contributing ‘savoury’ attributes; 3-methyl thiol propenol (‘meat’, ‘potato’ flavour) and ethyl-2-hydroxy-3-phenylpropanoate (‘goaty’, ‘smokey’). The hybrids also produced some solvent-extractable volatile compounds at different levels to each other, but, as opposed to the targeted volatile compounds, hybrid strain CxM4 generally produced the higher levels.

**Table 4:** Solvent-extractable volatile fermentation products of AWRI 838, CXM1 and CXM4 in Chardonnay wines.

			Peak Area X 10^4^		
R.T.	Compound Identity	Flavour Descriptor	AWRI838	CxM1	CxM4
16.51	Ethyl octanoate	Sweet, soap	215±8 a	216±17 a	220±30 a
16.67	Acetic acid	Vinegar	655±9 a	127±10 c	283±55 b
19.08	2-Methyl-tetrahydrothiophen-3-one	Blackberry, fruit berry	34±3 a	16±1 b	17±2 b
19.54	2.3-Butanediol	Cashew, rubber	195±27 a	208±39 a	179±42 a
19.67	2,6-Dimethyl-4-heptanol	Yeasty, fermented	144±13 a	84±2 b	104±9 b
20.45	Isobutyric acid	Cheese, rancid, sour	48±5 b	73±9 a	74±4 a
20.61	1,3-Butanediol	Butter	83±5 a	74±5 a	81±8 a
21.90	1,2-Butanolide	Smokey, hot	630±3 a,b	590±3 b	678±4 a
22.20	Butanoic acid	Cheese, rancid, sweaty	36±2 a	42±4 a	42±5 a
22.60	Ethyl decanoate	Floral, soap	137±4 a	98±13b	106±14 b
23.41	2-Methyl butanoic acid	Cheese, sour, rancid	58±2 b	88±16 a	45±6 b
23.48	Diethyl succinate	Fruity	194±9 b	243±2 a	248±20 a
24.05	Ethyl-9-decanoate	Sweet, pleasant	20±6 c	45±4 b	60±7 a
24.56	3-Methyl thiol propanol	Savoury, meat, potato	45±4 b	110±16 a	99±8 a
27.03	Ethyl 4-hydroxybutanoate	Sweet, pleasant	610±18 a	502±10 c	546±12 b
27.27	β-Phenyl acetate	Sweet, solvent	50±2 b	96±10 a	98±12 a
28.20	Hexanoic acid	Vinegar, fermented	258±27 a	229±52 a	284±14 a
29.77	2-Phenyl ethyl alcohol	Floral, rose	9619±153 c	18194±244 a	17115±461 b
33.05	Diethyl malate	Green, fruity, caramel	26±2 a	26±2 a	29±1 a
33.16	Unidentified		52±2 b	58±3 b	74±4 a
33.62	Octanoic acid	Harsh, rancid	642±30 a	650±41 a	676±64 a
38.05	3-Hydroxy-4-phenyl-2-butanone	Fruity, sweet, caramel	22±2 c	47±3 b	60±5 a
38.36	Unidentified		20±5 c	46±1 b	78±9 a
38.46	Ethyl-2-hydroxy-3-phenylpropanoate	Goaty, smokey	34±5 b	69±3 a	77±17 a
38.69	Decanoic acid	Fatty	372±32 a	260±41 a	320 95 a
40.02	9-Decenoic acid	Fruity, waxy	60±4 c	147±3 b	193±11 a
40.77	4-Vinyl phenol	Pharmaceutical	173±1 a,b	168±7 b	187±9 a
41.15	Ethyl hydrogen succinate	Fruit (mild)	183±43 a	329±26 a	215±24 a
42.13	Unidentified		61±12 a	55±3 a	56±3 a
53.51	4-Hydroxybenzene ethanol	Sweet floral, fruity	580±28 c	910±33 a	741±13b

Levels not connected by same letter are significantly different (p<0.05).

### Analyses of wine polyphenolics

Analysis of UV scan absorbance showed that both wines made with the hybrid strains contained higher levels of total phenolics, total hydroxycinnamic acids (HCA) and total flavonoid extracts, relative to the parent yeast-made wines (**Table 5**). Caffeic acid equivalents (CAE), a measure of non-flavonoid phenolics, was produced in higher amounts by both hybrid strains (110%). Hybrid strain CxM1 produced the highest level of catechin equivalents (CE), a measure of flavonoid phenolics, at 140%, with CxM4 producing 125% relative to the parent strain.

**Table 5:** Polyphenolic analysis of Chardonnay wines made by AWRI 838, CxM1 and CxM4 using UV Scan data: an index of Phenolic content.

	Total Phenolics (a.u.)	Total HCA (a.u.)	Flavonoid Extract (a.u.)	CAE (mg/L) (non-flavonoid)	CE (mg/L) (flavonoid)
AWRI838	3.75±0.03 c	3.90±0.02 b	1.15±0.01 c	43.3±0.26 b	80.7±0.82 c
CxM1	4.46±0.04 a	4.24±0.04 a	1.64±0.01 a	47.2±0.5 a	114.7±0.6 a
CxM4	4.24±0.06 b	4.20±0.04 a	1.44±0.03 b	46.7±0.5 a	100.6± 2.0 b

Levels not connected by same letter are significantly different (p<0.05).

## Discussion

The current downturn in the global economy continues to have a large impact on wine markets around the world. As winemakers vie for a share of this market, the need for product differentiation plays an important role in winemaking practices. Many winemakers desire the sensorial characteristics of complex aroma and flavour profiles of spontaneous fermentations, but are reluctant to risk a quality product to spoilage. Studies of spontaneous fermentations have identified a genetically diverse range of yeast, (*Saccharomyces cerevisiae* being but one), populations of which wax and wane over the duration of a fermentation [25], [26]. The metabolites produced by each yeast contribute to the myriad of flavours and aromas witnessed in the resultant wine [27]. Interspecific hybrid yeast have been shown to produce altered metabolite profiles relative to their *S. cerevisiae* wine yeast parent [17].

The use of a new robust *S. cerevisiae*-‘style’ wine yeast incorporating the genome of *S. cerevisiae* and a distant *Saccharomyces sensu stricto* species not associated with wine fermentation could potentially lead to wines with novel yeast-derived flavour-active metabolites. Indeed, traditional breeding techniques are used in the development of new yeast strains with altered phenotypic characteristics in brewing, breadmaking and winemaking industries [28], [29], [30], [31]. However, this approach requires sporulation of the wine yeast parent strain with subsequent segregation of traits, potentially leading to loss of robust winemaking properties in progeny [32]. Thus, for the current work, rare mating [33] was used to hybridise a diploid wine yeast with haploid spores of the non-wine yeast parent. (Previous studies have identified the triploid composition of natural, stable industrial/fermentation competent *Saccharomyces* hybrid yeast containing a diploid *S. cerevisiae* genome and a haploid non- *S. cerevisiae* genome [2], [34], giving a precedent to the generation of triploid interspecific hybrid yeast for this study.)

Although mating between spores of *S. cerevisiae* and *S. mikatae* has previously been performed to determine species boundaries [9], [35], no natural interspecific hybrids between these two species have been reported and no hybridisation events of diploid *S. cerevisiae* cells with *S. mikatae* spores have been reported previously.

Putative hybrids from successful rare mating events were confirmed using PCR-RFLP analysis of the ITS region within the rDNA tandem repeat on Chromosome XII. In addition, fluorescence flow cytometry analysis of CxM1 and CxM4 showed DNA fluorescent levels equivalent to a triploid genome, i.e. midway between levels displayed by the diploid and tetraploid control strains.

An assessment of parental phenotypic traits showed that all five hybrids from the *S. cerevisiae* x *S. mikatae* mating inherited traits from both parents: high temperature tolerance from the *S. cerevisiae* parent and low temperature tolerance from the *S. mikatae* parent. In addition, the hybrids also inherited from the *S. cerevisiae* wine yeast parent traits that are necessary for wine fermentation; the ability to grow on high sugar sources and tolerance to high ethanol levels. In fact, three of the five hybrid strains displayed transgressive phenotypes (hybrid vigor) with even stronger growth on high ethanol medium than their ethanol-tolerant *S. cerevisiae* parent.

The five hybrids differed in their abilities to tolerate stresses following inoculation into Chardonnay juice; there was an extended lag-phase prior to commencement of cell division for some hybrids. This is important because the practice of yeast inoculation of commercial wines requires the strain to quickly increase cell numbers in order outcompete indigenous, potentially spoilage, microorganisms. Yeast requiring an extended acclimatisation period in grape juice prior to the commencement of fermentation might compromise the quality of the resultant wine, hence hybrid strains showing this tendency are not suitable for commercial usage. On the other hand, two hybrid strains, (CxM1 and CxM4), showed a short lag-phase commensurate with the commercial wine yeast parent strain and were used for all subsequent in-depth wine fermentation analyses. The differences observed between individual hybrids, (growth in grape juice and wine chemical composition), may be attributable to heterozygosity of the *S. mikatae* diploid parent strain, sporulation of which would have led to spores carrying different combinations of alleles, resulting in triploid progeny containing identical *S. cerevisiae* genomes but differing *S. mikatae* alleleic content.

Basic fermentation chemistry analysis of the wines showed that all five hybrid strains were all able to convert sugars to ethanol, with resultant wines containing similar ethanol levels to the *S. cerevisiae* parent-made wines. Differences to note in the hybrid-made wines were, for all hybrids, an increase in glycerol production and a decrease in acetic acid production relative to the wine yeast parent. Glycerol is known to add to the sweetness of wine [36] and, due to its viscous nature, contributes to the smoothness and overall body of a wine [37], [38], while acids greatly influence the taste of wines, contributing to the crispness of the palate. However, acetic acid, with the non-desirable volatile and odorous aroma of ‘vinegar’ is of particular concern to winemakers. Wine yeast strains producing higher levels of glycerol while at the same time producing low, or undetectable, concentrations of acetic acid would greatly assist winemakers in improving the quality of their wines.

On the other hand, chemical analysis showed that some hybrid-made wines contained residual sugar in the form of fructose. The inactivation of sugar transport systems in yeast cells during alcoholic fermentation [39] and alterations to the glucose-fructose ratio of the fermenting must [40] often lead to sluggish or stuck fermentations, with the resultant wine having residual fructose. Yeast strains developed for the wine industry should to be free from potential fermentation problems, thus hybrid strains producing wines with residual fructose were considered to be unsuitable for further investigation.

The two hybrid strains, (CxM1 and CxM4), exhibiting problem-free, robust fermentation properties were chosen for further study. The chromosomal complement of hybrid strain CxM1 was investigated using a-CGH with results indicating that a complete set of chromosomes from each parent species exist in the hybrid genome. The hybrid showed lower fluorescence intensities of *S. mikatae* probes, relative to the *S. mikatae* parent, whereas intensities to *S. cerevisiae* probes were similar to that of the *S. cerevisiae* parent. This implies a haploid *S. mikatae* chromosomal content, which is in keeping with the flow cytometry results (triploid DNA content), indicating that the hybrid was formed when diploid *S. cerevisiae* cells mated with spores from *S. mikatae*.

The varied fluorescence intensity of bound *S. cerevisiae* probes in the microarray can be attributed to the polymorphic DNA sequence of the wine yeast parent strain, AWRI838 [41], resulting in diverse binding affinities to the probes designed to the S288c *S. cerevisiae* genome.

Initially, genetic stability of hybrid strains was assessed by the retention of ribosomal DNA from each parent. Plant studies have shown that changes in rDNA (loss or silencing of rDNA from one parental species) occurs at the incipient stages of evolution of interspecific hybrids [42], [43], [44]. The two hybrid strains chosen for further investigation (CxM1 and CxM4) had relatively stable genomes under the stressful fermentation conditions, (low pH and high sugar early in fermentation followed by high levels of ethanol in the later stages), with end-of-fermentation isolates revealing a loss of *S. mikatae* rDNA in only one of a total of 300 isolates analysed.

Subsequently, genomic analysis on end-of-fermentation isolates, targeting each of the sixteen chromosomes from both parental species was carried out. This was followed by phenotypic analysis to determine the retention of essential fermentation traits. A small number of isolates, (4% of CxM1 and 8% of CxM4), showed minor chromosomal alterations, with loss of one or both arms of a single chromosome from the *S. mikatae*-parent genome. In the first instance, primers were designed to a region towards the telomere of the long arm of each chromosome and if genomic loss was identified, then the short arm of the chromosome was investigated. No loss of *S. cerevisiae* chromosomal genome was detected in any isolate and fluorescence flow cytometry detected no loss of overall ploidy. However, there may be losses or duplications not detected by the methods used in this study. Importantly, the fermentation properties of tolerance to high sugar and ethanol levels were retained in all isolates, even those with partial loss of the *S. mikatae* genome. Studies have shown that genome instability can occur in tetraploid strains of *S. cerevisiae* [45] whereas polyploid *S. cerevisiae* interspecific hybrids have been shown to be more stable than polyploid *S. cerevisiae* intraspecific hybrids [46]. However, both studies involved yeast cell replication over a large number of generations and/or repeated re-pitching of cells into stressful environs. The modern winemaking practice of inoculation with an Active Dried Yeast preparation made from original stock cultures requires yeast to undergo only a maximum of seven to eight replication events during the course of fermentation, hence minimising the risk of large-scale instability impacting on fermentation performance and wine quality. Wine yeast are not re-pitched from one fermentation to the next.

Importantly, from a winemaking perspective, desirable transgressive phenotypes were apparent in CxM1 and CxM4 hybrids in the form of increased concentrations of secondary metabolites. Chardonnay wines produced using these hybrids showed differences in concentrations in a number of the target volatile metabolite compounds, relative to wine made using the parent *S. cerevisiae* wine yeast. Hybrid strain CxM1 produced higher concentrations in a number of compounds associated with flavours of ‘fruity’, ‘banana’, ‘floral’ and ‘sweet perfume’. Increasing the concentration of a flavour or aroma compound can lead to an increased sensory impact of that particular compound, but may also lead to the masking of other flavours or aromas [47]. Conversely, although the second hybrid strain, (CxM4), produced wines with a greater number of compounds at different concentrations to what was present in the parent-made wine, all but one of the differences resulted in a decrease in concentration, with only 2-phenylethyl acetate (‘floral’ aroma) showing a two-fold increase. A positive side to the production of lower metabolite concentrations is that this yeast also produced lower levels of the three compounds analysed with the non-desirable aroma of nail polish. Lowering the concentration of a compound, particularly compounds with a negative sensory attribute, impacts not only on the compound concerned, but may also un-mask other flavours and aromas [47].

Chemical analysis of the solvent-extractable volatile portion of the wines also revealed differences in levels of flavour active metabolites. The hybrid yeast-made wines showed significantly higher levels of a number of compounds, including isobutyric acid (‘sour’, ‘cheese’), 3-methyl thiol propanol (‘meat’, ‘potato’) and ethyl-2-hydroxy-3-phenylpropanoate (‘goaty’, ‘smokey’), all which contribute savoury attributes that potentially add complexity to the overall flavour profile of these wines. Three solvent-extracted volatile compounds remain unidentified, two of which were produced at higher levels by the hybrid yeast and this may indicate that the *S. mikatae* parent is contributing novel metabolites, not previously recognised, to the wines. Of interest also, is that two identified compounds produced at higher levels by the *S. cerevisiae* x *S. mikatae* hybrids have been shown to be generated in wine in high levels by non-*Saccharomyces cerevisiae* species: isobutyric acid, *Torulaspora delbrueckii* [48] and 2-phenyl ethyl alcohol, *Kluyveromyces lactis* [49].

Polyphenols contribute to sensory properties in wine. Grape and wine phenolic compounds can be divided into two groups; non-flavonoids and flavonoids. The primary class of non-flavonoids in white wine is the hydroxycinnamates (HCA), with esters of caffeic acid being the most abundant [50]. HCAs are potent antioxidants and have been shown to be involved in the prevention of browning of musts and wines [51] while catechins, a major class of flavonoids, are known for their bitterness [52]. In the current work, polyphenolic content was assessed by spectral evaluation and estimations of non-flavonoid and flavonoid content were derived by using extinction co-efficients [23], [24]. CxM1 and CxM4 produced wines with slightly higher levels of flavonoid and non-flavonoid content. It has been shown that differences in the concentrations of hydroxycinnamic derivatives constitute an important factor in browning, with the proportion of tartaric esters of caffeic acid, *p*-coumaric acid and ferulic acid playing important roles [51]. Both of the hybrid strains produced wines with higher concentrations of phenolics, (including total hydroxycinnamates), relative to wine produced by the wine yeast parent, potentially leading to different impacts on browning.

In conclusion, a new breed of interspecific wine yeast has been developed that incorporates the genomes of *S. cerevisiae* and *S. mikatae*, the latter of which has not previously been associated with wine fermentation. Whilst there are numerous natural *S. cerevisiae* x *Saccharomyces spp.* interspecific hybrids reported in the literature, no natural *S. cerevisiae* x *S. mikatae* hybrids have been isolated. The evolutionary distance between these two yeasts is considerable (they share only 73% of overall DNA sequence homology), therefore it was deemed to be a good candidate for the introduction of novel metabolic outputs to shape wine sensory characteristics. This proved to be the case; chemical analyses of wines made using *S. cerevisiae* x *S. mikatae* hybrids confirmed that the presence of a *S. mikatae* genome impacted favourably on the production of flavour-active volatile fermentation metabolites, potentially producing complex wines akin to spontaneous ferments. The safeguard of an inoculated ferment while providing complexity to their wines assists winemakers by providing additional tools to develop new wine styles.

## Supporting Information

Figure S1 **Genetic stability of fermentation isolates from CxM1 and CxM4 using chromosomal targeted PCR-RFLP.** Figure S1a. CxM1 fermentation isolates. First gel Chromosome I left arm, second gel Chromosome II right arm, third gel Chromosome III right arm, fourth gel Chromosome IV right arm, fifth gel Chromosome V left arm, sixth gel Chromosome V right arm, seventh gel Chromosome VI left arm, eighth gel, Chromosome VII left arm and ninth gel Chromosome VIII left arm. In all gels; Lane 1 100 bp ladder, lane 2 AWRI838, Lane 3 NCYC2888, lane 4 DNA from both parents, lane 5 Hybrid CxM1, lanes 6 to 55 isolates 1 to 50. Figure S1b. CxM1 fermentation isolates continued. First gel Chromosome IX left arm, second gel Chromosome X left arm, third gel Chromosome X right arm, fourth gel Chromosome XI left arm, fifth gel Chromosome XII left arm, sixth gel Chromosome XII right arm, seventh gel Chromosome XIII right arm and eighth gel Chromosome XV left arm. In all gels; Lane 1 100 bp ladder, lane 2 AWRI838, Lane 3 NCYC2888, lane 4 DNA from both parents, lane 5 Hybrid CxM1, lanes 6 to 55 isolates 1 to 50. Figure S1c. CxM4 fermentation isolates. First gel Chromosome I left arm, second gel Chromosome II right arm, third gel Chromosome III right arm, fourth gel Chromosome IV right arm, fifth gel Chromosome VI left arm, sixth gel Chromosome VII left arm, seventh gel Chromosome VIII left arm, eighth gel and Chromosome IX right arm. In all gels; Lane 1 100 bp ladder, lane 2 AWRI838, Lane 3 NCYC2888, lane 4 DNA from both parents, lane 5 Hybrid CxM4, lanes 6 to 55 isolates 1 to 50. Figure S1d. CxM4 fermentation isolates. First gel Chromosome XI left arm, second gel Chromosome XIII right arm, third gel Chromosome XIV right arm, fourth gel Chromosome XV left arm, fifth gel Chromosome XVI left arm and sixth gel Chromosome XVI right arm. In all gels; Lane 1 100 bp ladder, lane 2 AWRI838, Lane 3 NCYC2888, lane 4 DNA from both parents, lane 5 Hybrid CxM4, lanes 6 to 55 isolates 1 to 50.
(PDF)

Figure S2 **Fluorescence flow cytometry analysis of hybrid CxM1 post-fermentation isolates.** Figure S2a. Row 1 Strains left to right; BY4742 (haploid), BY4743 (diploid), 53–7 (tetraploid), CxM1 (AWRI2526). CxM1 isolates left to right; Row 2 Isolate 1–5, Row 3 Isolate 5–10, Row 4 Isolate 11–15. Figure S2b. CxM1 isolates left to right; Row 1 Isolate 16–20, Row2, Isolate 21–25, Row 3 Isolate 26–30, Row 4 Isolate 31–35. Figure S2c. CxM1 isolates left to right; Row 1, Isolate 36–40, Row 2 Isolate 41–45, Row 3 Isolate 46–50.
(PDF)

Figure S3 **Phenotypic assessment assay plates of CxM1 post-fermentation isolates.** Figure S3a. Plates left to right; YEPD at temperature 22°C, YEP 25% glucose, YEPD 14% ethanol. Strains are plated in columns at 10 fold serial dilutions from top to bottom in two sections of the plate. Top section left to right; AWRI 838 (*Sc*), NCYC2888 (*Sm*), CxM1, CxM1 isolates 1–5. Bottom section left to right; CxM1 isolates 6–13. Figure S3b. Plates left to right; YEPD at temperature 22°C, YEP 25% glucose, YEPD 14% ethanol. Strains are plated in columns at 10 fold serial dilutions from top to bottom in two sections of the plate. Top section left to right; AWRI 838 (*Sc*), NCYC2888 (*Sm*), CxM1, CxM1 isolates 14–18. Bottom section left to right; CxM1 isolates 19–26. Figure S3c. Plates left to right; YEPD at temperature 22°C, YEP 25% glucose, YEPD 14% ethanol. Strains are plated in columns at 10 fold serial dilutions from top to bottom in two sections of the plate. Top section left to right; AWRI 838 (*Sc*), NCYC2888 (*Sm*), CxM1, CxM1 isolates 27–31. Bottom section left to right; CxM1 isolates 32–39. Figure S3d. Plates left to right; YEPD at temperature 22°C, YEP 25% glucose, YEPD 14% ethanol. Strains are plated in columns at 10 fold serial dilutions from top to bottom in two sections of the plate. Top section left to right; AWRI 838 (*Sc*), NCYC2888 (*Sm*), CxM1, CxM1 isolates 40–44. Bottom section left to right; CxM1 isolates 45–50.
(PDF)
