# Supplementary figures and images for: Introducing a New Breed of Wine Yeast: Interspecific Hybridisation between a Commercial Saccharomyces cerevisiae Wine Yeast and Saccharomyces mikatae
Source: PLoS One. 2013 Apr 17;8(4):e62053. doi: 10.1371/journal.pone.0062053 (PMC3629166; doi:10.1371/journal.pone.0062053)

Supporting Figure S1a

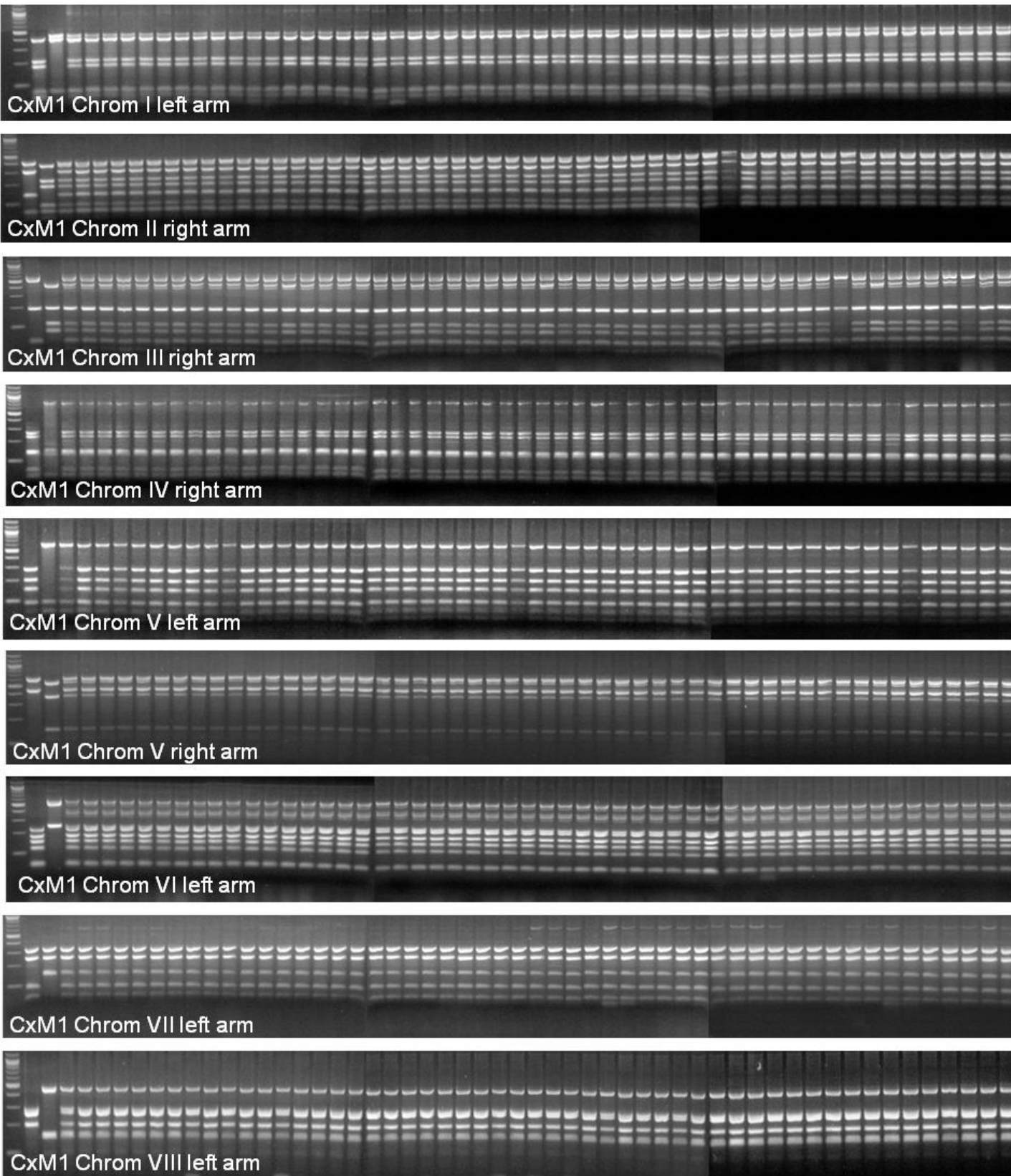

Supporting Figure S1b

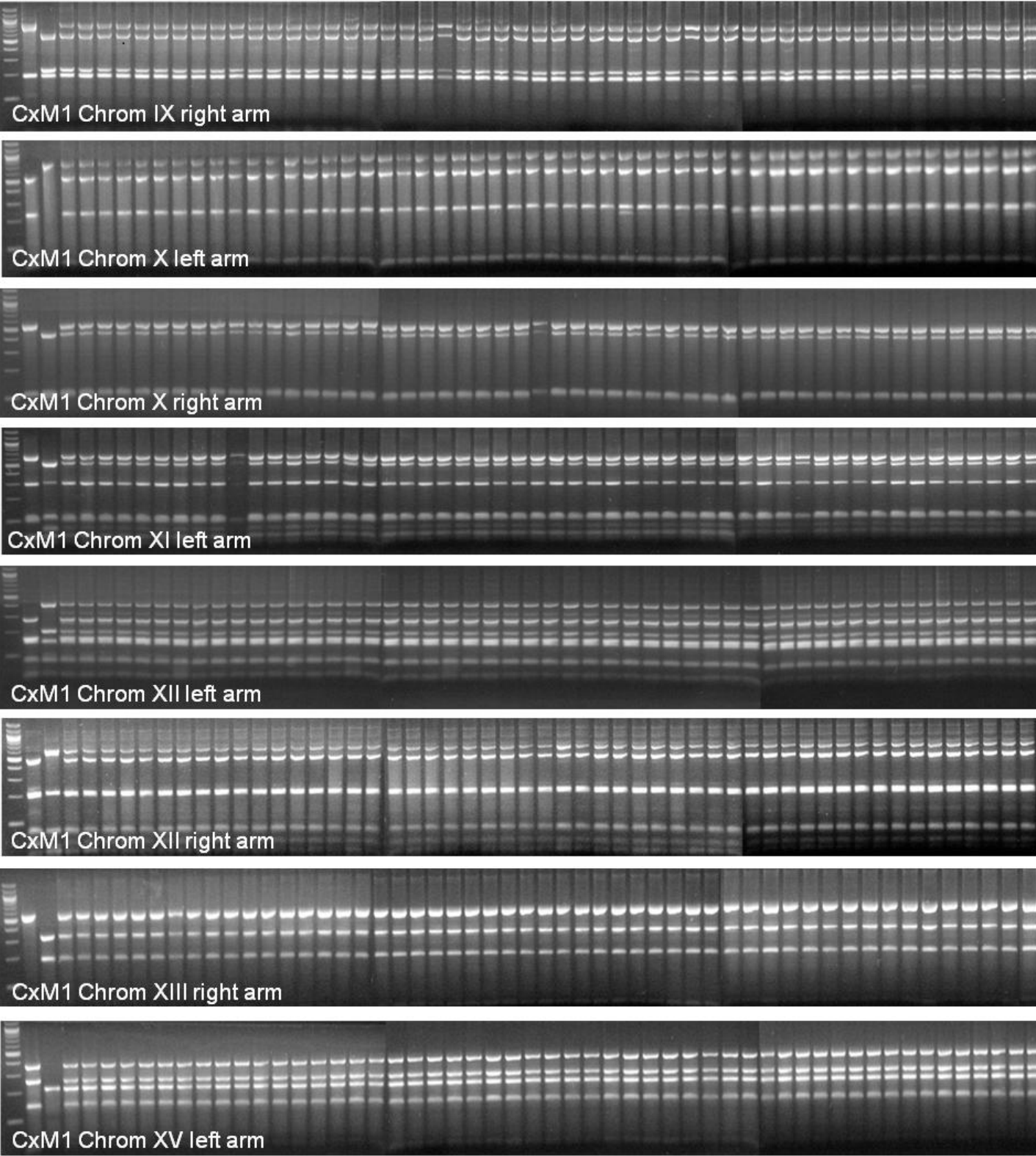

Supporting Figure S1c

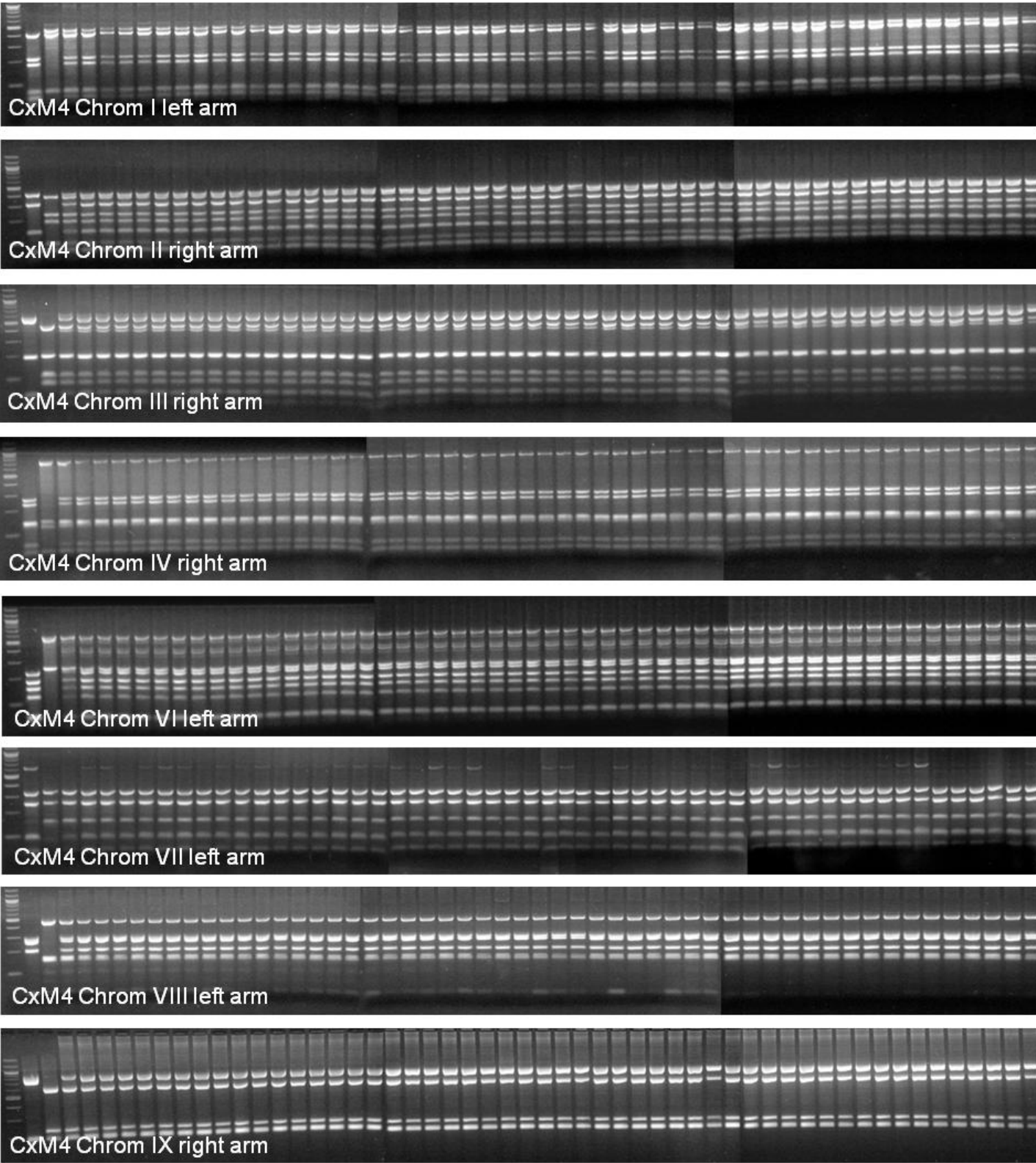

Supporting Figure S1d

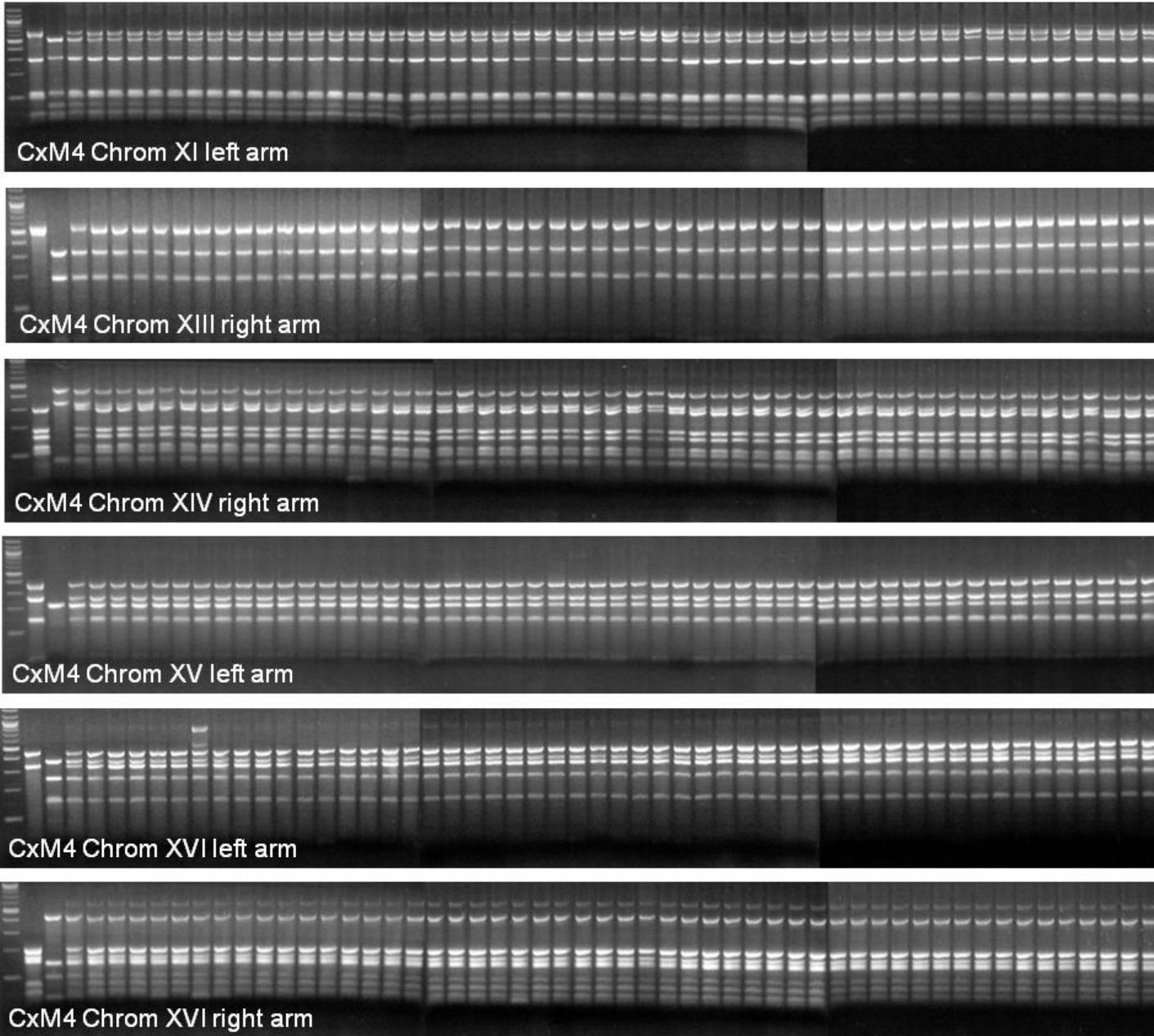

Supplement: Figure S1 — Genetic stability of fermentation isolates from CxM1 and CxM4 using chromosomal targeted PCR-RFLP. Figure S1a. CxM1 fermentation isolates. First gel Chromosome I left arm, second gel Chromosome II right arm, third gel Chromosome III right arm, fourth gel Chromosome IV right arm, fifth gel Chromosome V left arm, sixth gel Chromosome V right arm, seventh gel Chromosome VI left arm, eighth gel, Chromosome VII left arm and ninth gel Chromosome VIII left arm. In all gels; Lane 1 100 bp ladder, lane 2 AWRI838, Lane 3 NCYC2888, lane 4 DNA from both parents, lane 5 Hybrid CxM1, lanes 6 to 55 isolates 1 to 50. Figure S1b. CxM1 fermentation isolates continued. First gel Chromosome IX left arm, second gel Chromosome X left arm, third gel Chromosome X right arm, fourth gel Chromosome XI left arm, fifth gel Chromosome XII left arm, sixth gel Chromosome XII right arm, seventh gel Chromosome XIII right arm and eighth gel Chromosome XV left arm. In all gels; Lane 1 100 bp ladder, lane 2 AWRI838, Lane 3 NCYC2888, lane 4 DNA from both parents, lane 5 Hybrid CxM1, lanes 6 to 55 isolates 1 to 50. Figure S1c. CxM4 fermentation isolates. First gel Chromosome I left arm, second gel Chromosome II right arm, third gel Chromosome III right arm, fourth gel Chromosome IV right arm, fifth gel Chromosome VI left arm, sixth gel Chromosome VII left arm, seventh gel Chromosome VIII left arm, eighth gel and Chromosome IX right arm. In all gels; Lane 1 100 bp ladder, lane 2 AWRI838, Lane 3 NCYC2888, lane 4 DNA from both parents, lane 5 Hybrid CxM4, lanes 6 to 55 isolates 1 to 50. Figure S1d. CxM4 fermentation isolates. First gel Chromosome XI left arm, second gel Chromosome XIII right arm, third gel Chromosome XIV right arm, fourth gel Chromosome XV left arm, fifth gel Chromosome XVI left arm and sixth gel Chromosome XVI right arm. In all gels; Lane 1 100 bp ladder, lane 2 AWRI838, Lane 3 NCYC2888, lane 4 DNA from both parents, lane 5 Hybrid CxM4, lanes 6 to 55 isolates 1 to 50. (PDF) [file pone.0062053.s001.pdf]

Supporting Figure S2a

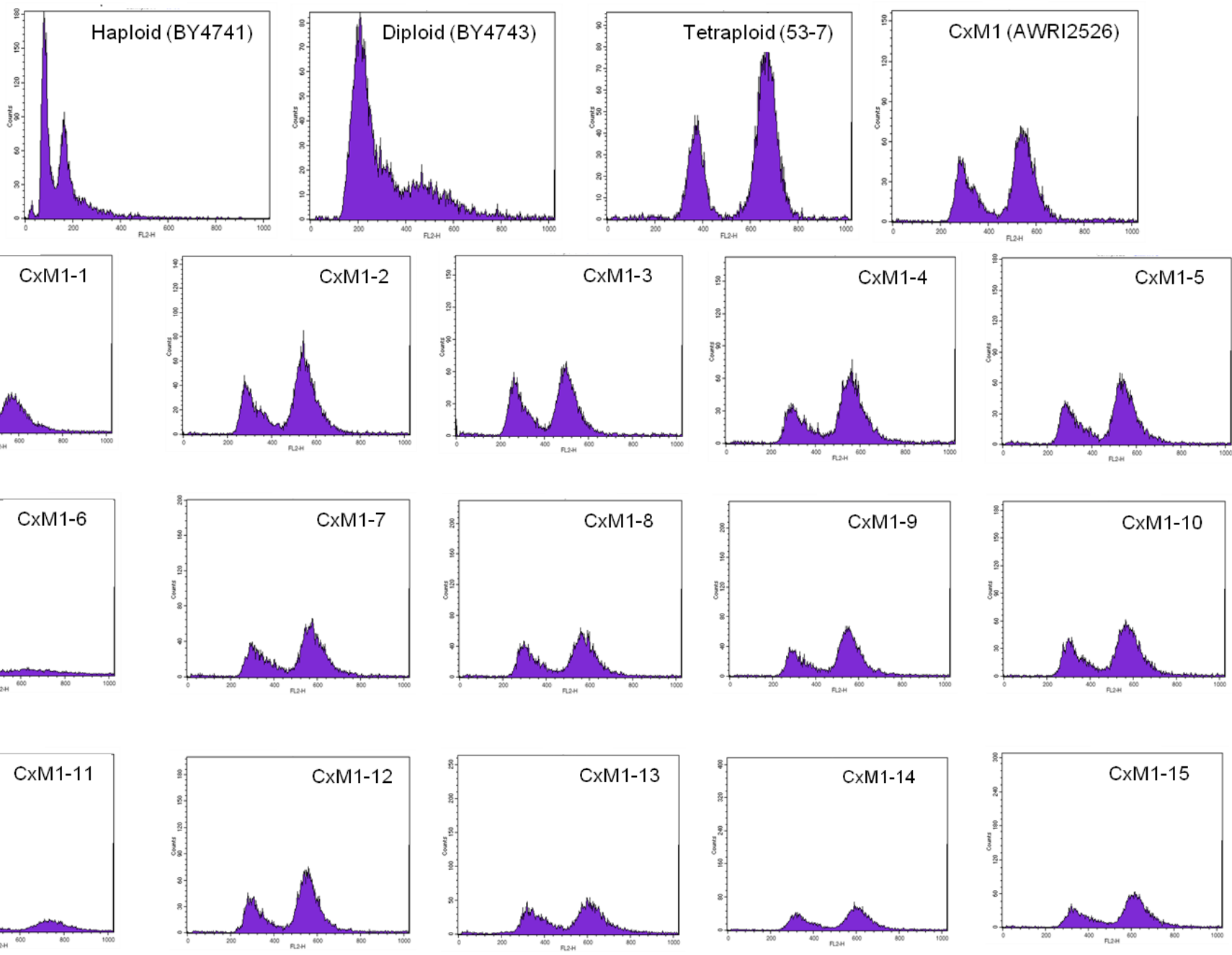

Supporting Figure S2b

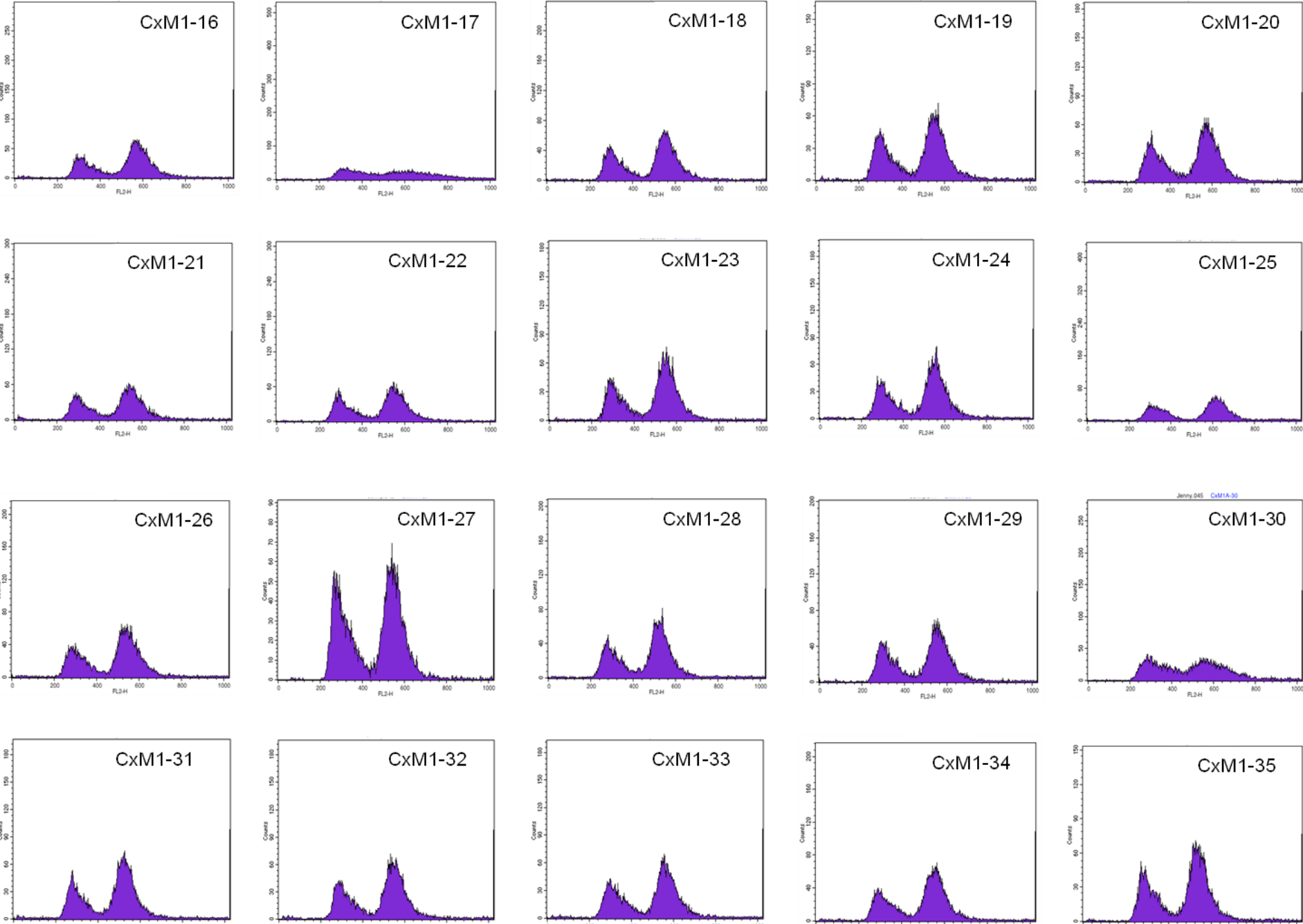

Supporting Figure S2c

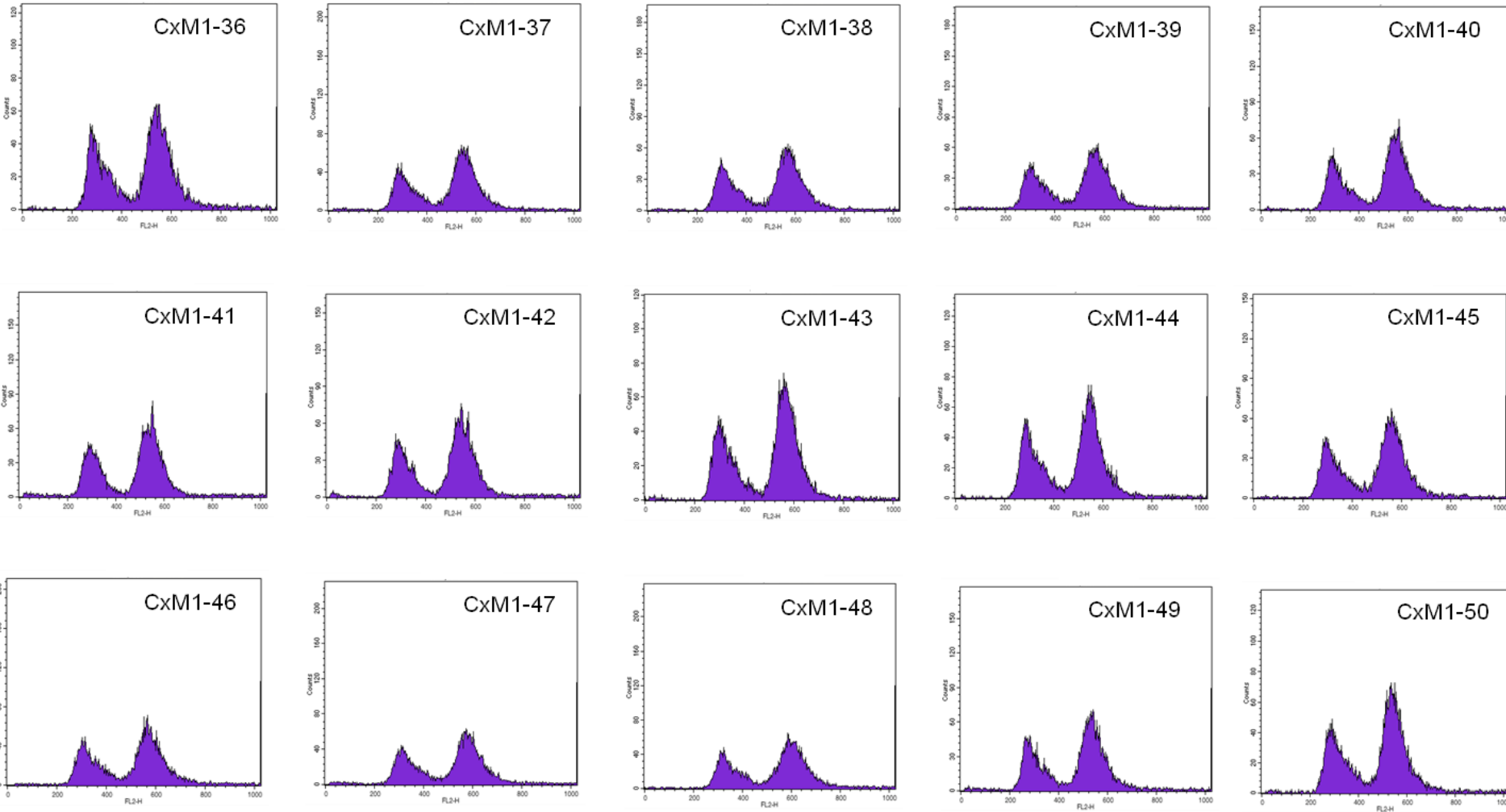

Supplement: Figure S2 — Fluorescence flow cytometry analysis of hybrid CxM1 post-fermentation isolates. Figure S2a. Row 1 Strains left to right; BY4742 (haploid), BY4743 (diploid), 53–7 (tetraploid), CxM1 (AWRI2526). CxM1 isolates left to right; Row 2 Isolate 1–5, Row 3 Isolate 5–10, Row 4 Isolate 11–15. Figure S2b. CxM1 isolates left to right; Row 1 Isolate 16–20, Row2, Isolate 21–25, Row 3 Isolate 26–30, Row 4 Isolate 31–35. Figure S2c. CxM1 isolates left to right; Row 1, Isolate 36–40, Row 2 Isolate 41–45, Row 3 Isolate 46–50. (PDF) [file pone.0062053.s002.pdf]
