# Supplementary material for: Introducing a New Breed of Wine Yeast: Interspecific Hybridisation between a Commercial Saccharomyces cerevisiae Wine Yeast and Saccharomyces mikatae
Source: PLoS One. 2013 Apr 17;8(4):e62053. doi: 10.1371/journal.pone.0062053 (PMC3629166; doi:10.1371/journal.pone.0062053)

Supporting Figure S3a

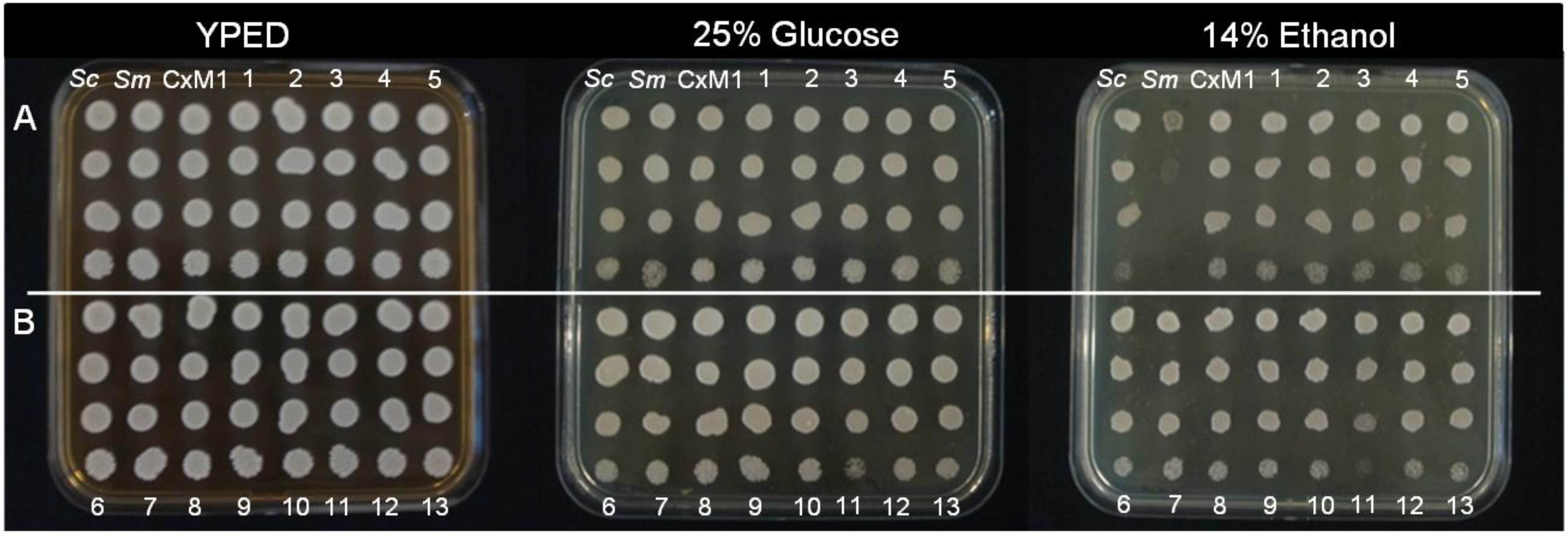

Supporting Figure S3b

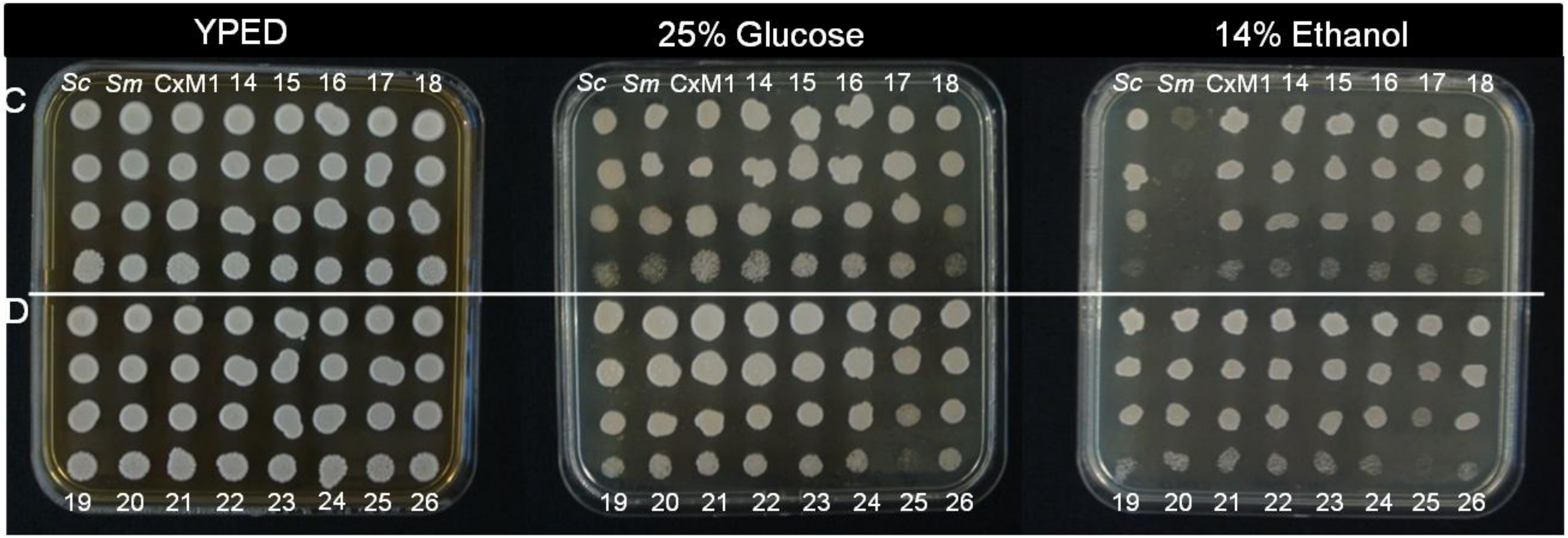

Supporting Figure S3c

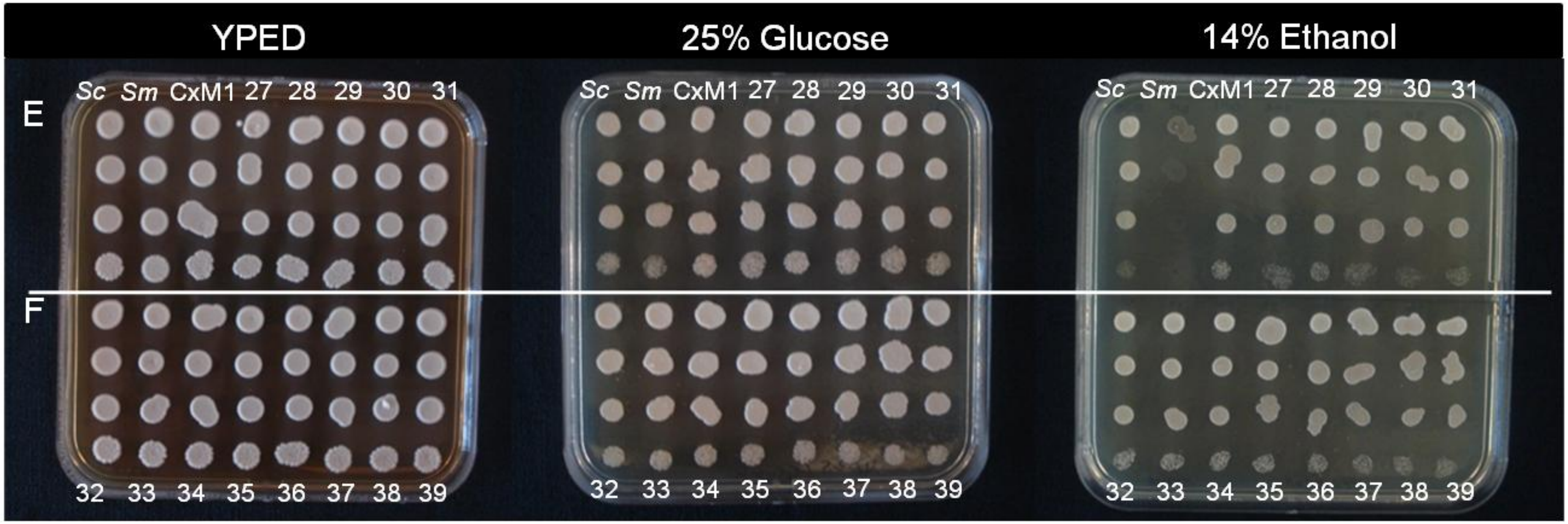

Supporting Figure S3d

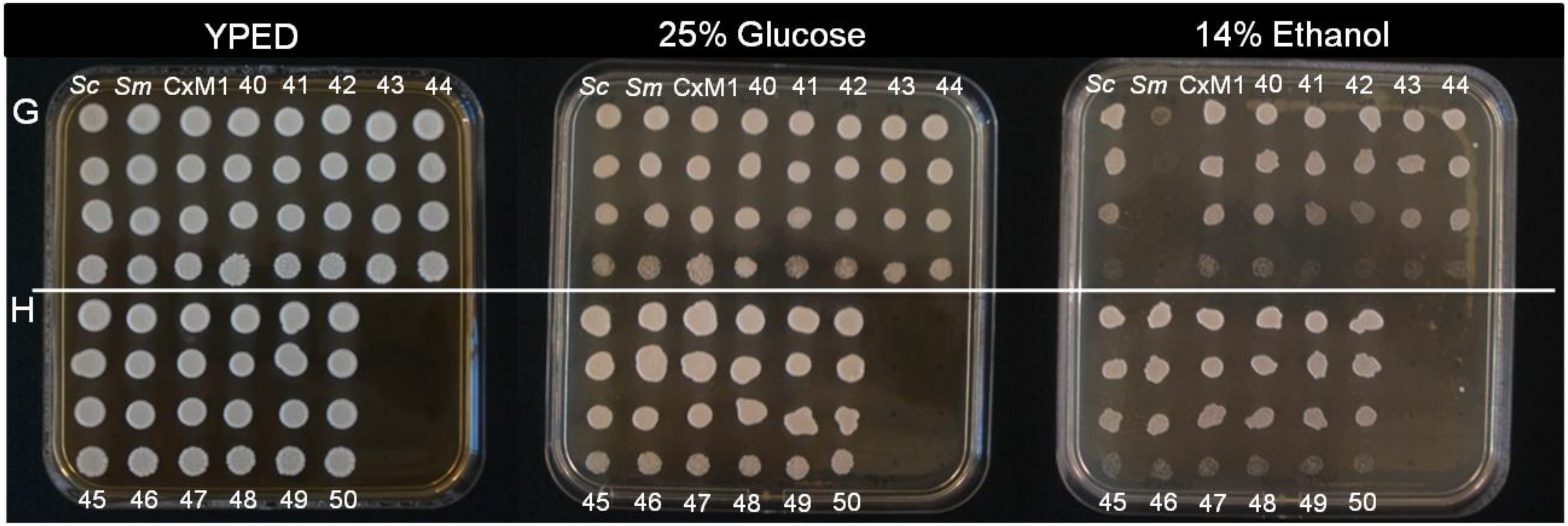

Supplement: Figure S3 — Phenotypic assessment assay plates of CxM1 post-fermentation isolates. Figure S3a. Plates left to right; YEPD at temperature 22°C, YEP 25% glucose, YEPD 14% ethanol. Strains are plated in columns at 10 fold serial dilutions from top to bottom in two sections of the plate. Top section left to right; AWRI 838 (Sc), NCYC2888 (Sm), CxM1, CxM1 isolates 1–5. Bottom section left to right; CxM1 isolates 6–13. Figure S3b. Plates left to right; YEPD at temperature 22°C, YEP 25% glucose, YEPD 14% ethanol. Strains are plated in columns at 10 fold serial dilutions from top to bottom in two sections of the plate. Top section left to right; AWRI 838 (Sc), NCYC2888 (Sm), CxM1, CxM1 isolates 14–18. Bottom section left to right; CxM1 isolates 19–26. Figure S3c. Plates left to right; YEPD at temperature 22°C, YEP 25% glucose, YEPD 14% ethanol. Strains are plated in columns at 10 fold serial dilutions from top to bottom in two sections of the plate. Top section left to right; AWRI 838 (Sc), NCYC2888 (Sm), CxM1, CxM1 isolates 27–31. Bottom section left to right; CxM1 isolates 32–39. Figure S3d. Plates left to right; YEPD at temperature 22°C, YEP 25% glucose, YEPD 14% ethanol. Strains are plated in columns at 10 fold serial dilutions from top to bottom in two sections of the plate. Top section left to right; AWRI 838 (Sc), NCYC2888 (Sm), CxM1, CxM1 isolates 40–44. Bottom section left to right; CxM1 isolates 45–50. (PDF) [file pone.0062053.s003.pdf]
